# Perceptual integration of bodily and facial emotion cues in chimpanzees and humans

**DOI:** 10.1093/pnasnexus/pgae012

**Published:** 2024-01-18

**Authors:** Raphaela Heesen, Yena Kim, Mariska E Kret, Zanna Clay

**Affiliations:** Department of Psychology, Durham University, Durham DH1 3LE, UK; Centre for the Advanced Study of Collective Behaviour, University of Konstanz, Konstanz 78464, Germany; Institute of Psychology, Cognitive Psychology Unit, Leiden University, Leiden 2333 AK, The Netherlands; Institute of Psychology, Cognitive Psychology Unit, Leiden University, Leiden 2333 AK, The Netherlands; The Leiden Institute for Brain and Cognition, Leiden University, Leiden 2300 RC, The Netherlands; Department of Psychology, Durham University, Durham DH1 3LE, UK

**Keywords:** eye-tracking, perception, emotional body language, attention, communication

## Abstract

For highly visual species like primates, facial and bodily emotion expressions play a crucial role in emotion perception. However, most research focuses on facial expressions, while the perception of bodily cues is still poorly understood. Using a novel comparative priming eye-tracking design, we examined whether our close primate relatives, the chimpanzees (*Pan troglodytes*), and humans infer emotions from bodily cues through subsequent perceptual integration with facial expressions. In experiment 1, we primed chimpanzees with videos of bodily movements of unfamiliar conspecifics engaged in social activities of opposite valence (*play* and *fear*) against neutral control scenes to examine attentional bias toward succeeding congruent or incongruent facial expressions. In experiment 2, we assessed the same attentional bias in humans yet using stimuli showing unfamiliar humans. In experiment 3, humans watched the chimpanzee stimuli of experiment 1, to examine cross-species emotion perception. Chimpanzees exhibited a persistent *fear-related* attention bias but did not associate bodily with congruent facial cues. In contrast, humans prioritized conspecifics' congruent facial expressions (matching bodily scenes) over incongruent ones (mismatching). Nevertheless, humans exhibited no congruency effect when viewing chimpanzee stimuli, suggesting difficulty in cross-species emotion perception. These results highlight differences in emotion perception, with humans being greatly affected by fearful and playful bodily cues and chimpanzees being strongly drawn toward fearful expressions, regardless of the preceding bodily priming cue. These data advance our understanding of the evolution of emotion signaling and the presence of distinct perceptual patterns in hominids.

**Significance Statement**

Emotion communication is vital for the navigation of social life. In primates, emotional expressions are tightly linked to the visual sensory channel, including facial and bodily movements. Despite the pertinence of visual communication in hominids, knowledge of how humans and their closest ape cousins compare in the perception of bodily and facial emotion cues is largely missing. Using a novel priming eye-tracking design, our work shows that chimpanzees and humans differ in their cross-modal integration of bodily and facial emotion cues, providing evidence of cross-species differences in emotion perception. While chimpanzees exhibited a persistent *fear-related* attention bias, humans readily integrated facial and bodily information. These data and developed methodology provide new empirical avenues for the field of comparative affective science, as they enable systematic species comparisons on emotion perception.

## Introduction

The navigation of social interactions implies a prompt and accurate reading of others' emotional states. Human emotion expression and perception rely on cross-modal displays, involving audio-visual-tactile and even olfactory channels (1, 2). Even before their first birthday, human infants recognize emotions by integrating facial and vocal cues to make informed decisions in ambiguous situations (3–7). Evidence also shows that human emotional expressions are rarely produced in isolation, but rather alongside multiple contextual cues related to language use, postures, and the surrounding (social) setting (8–13). These lines of evidence suggest a deep biological basis of multimodal and context-dependent emotion perception, warranting a comparative analysis to identify hallmarks of this complex perceptual system in our closest ape relatives. However, comparative evidence in chimpanzees and bonobos (*Pan paniscus*) was long missing and only recently started to emerge, coinciding with a rise in comparative psychophysiological techniques like eye-tracking and computerized paradigms (14, 15). Due to the close phylogenetic link between *Homo* and *Pan*, comparative evidence with chimpanzees and bonobos is crucial to understand how our affective communication system evolved from ancestral primate roots.

Although comparative research has provided evidence regarding how nonhuman primates, notably apes, *express* purported emotional states via communicative signals (1, 16–18), evidence of how they *perceive* such signals is still scarce (but see for instance 15, 19). Two studies focusing on chimpanzees and bonobos suggest that these species prioritize attention toward emotional compared to neutral stimuli (static images (15), dynamic video scenes (19), but see Ref. (20)) in similar ways to humans (21). Chimpanzees can also better remember and recognize stimuli showing aggressive compared to relaxed chimpanzees (body and face), even when presented as static images (22). Specifically, research relying on match-to-sample paradigms shows that chimpanzees can recognize distinct facial emotion expressions from static images (23) and discriminate between expressions of differing emotional valence (24). In similar match-to-sample tasks, chimpanzees can recognize and discriminate static images of facial expressions *and* match these to corresponding dynamic video material, providing the basis of stimuli use also employed in our current eye-tracking study (24, 25). With no prior training, chimpanzees can spontaneously match emotional video scenes (e.g. a conspecific being injected with a needle) with static images of facial expressions based on emotional meaning, even when the still images are in black and white (25). Beyond apes, monkeys like several species of macaques (e.g. *Macaca nigra* (26) and *Macaca mulatta* (27)) and tufted capuchins monkeys (*Sapajus apella* (28)) appear to discriminate emotional facial expressions, suggesting that emotion recognition is widespread in primates (see for review, Ref. (29)).

However, in comparison to other signal components, notably facial expressions, the body still appears to be a particularly understudied—though important—component of emotion perception (30). In close-range settings, facial or vocal expressions are naturally integrated with bodily cues in humans, and perceived as a “single person unit” (30 p. 15). Despite this and the fact that most primates are highly attuned to visual sensory modality (31–34), bodily expressions of emotions (and especially perception thereof) remain little understood, both in humans and nonhuman primates, compared to vocal or facial expressions (35, 36). Kret et al. (1) suggest that humans and most other primates possess a rich repertoire of visual emotion expressions, notably bodily expressions. The visual sensory modality thus appears to represent a cornerstone of primate affective communication. In humans, emotion research is heavily biased by evidence on facial expressions, though researchers recently began to recognize the impact of bodily movements on emotion perception (13, 36–40).

In terms of evidence on bodily perception in apes, one computerized task examined attention in chimpanzees toward naturalistic bodily movie scenes using looking time (19). While keeping facial and vocal information masked, the authors presented whole-body expressions in scenes of social interactions between conspecifics across a range of categories, including neutrality, conflict, play, and generic excitement. The study revealed that, similar to humans, chimpanzees looked longer toward bodily scenes associated with fear as compared to any other category (Ref. (19), but see Ref. (20) for lack of such a bodily emotion bias in a dot-probe paradigm using still images). This reported “fear-related” bias in chimpanzee bodily emotion perception is mostly in line with reported biases in humans, which shows longer looking times at fearful compared to affiliative or neutral expressions, both in terms of facial as well as bodily displays (Refs. (36, 39, 40), but see Ref. (41) for variation depending on the paradigm). Respective findings from bonobos are mixed. In a “dot-probe” study investigating immediate attentional bias, bonobos were specifically drawn toward scenes depicting sex, grooming, and yawning, as opposed to scenes focusing on feeding or distressed bonobos, suggesting a tendency toward protective and affiliative emotions (Ref. (15), but see Ref. (42) for different findings using the same bonobo group). The study also revealed faster reaction times for emotional over neutral expressions (15), an emotion bias that appears to only hold when stimuli of unfamiliar bonobos are used, yet not those showing familiar bonobos, as demonstrated by a recent dot-probe study with the same subjects (43).

To advance knowledge on how apes perceive bodily emotion cues and integrate this with information from facial expressions, we initially preregistered a priming eye-tracking study (e.g. 44) focusing on chimpanzees and bonobos (https://aspredicted.org/yq6bx.pdf). Our aim was to show bodily priming scenes of different valence categories (details below) and to subsequently measure attention to static images of facial emotion expressions, which were either congruent or incongruent with the preceding scenes. Vocal and facial cues were otherwise masked in the bodily priming stimuli (following 19). Since the COVID pandemic largely restricted access to relevant field sites of bonobos, we were only able to test chimpanzees in the current study. To provide a validation of the experimental design and cross-species comparison, we instead included an additional investigation of emotion perception in human participants. To this end, our study was composed of three consecutive experiments containing the same design but different stimuli and participants.

In experiment 1, chimpanzees viewed emotional interaction scenes of unfamiliar conspecifics (*play* and *fear* primers) and two different neutral control scenes (*fish tank* and conspecific *resting* primers), which were followed by image pairs portraying either an affiliative *play face* expression (next to a neutral expression) or a fear-related *bared-teeth* facial expression (next to a neutral expression). In experiment 2, we validated the findings of experiment 1, insofar as human participants viewed similar emotional interaction scenes of unfamiliar human conspecifics (*play* and *fear* primers) and neutral control scenes (*fish tank* and conspecific *resting* primers), likewise followed by image pairs portraying either a *smiling* facial expression (next to a neutral expression) or a *fearful* facial expression (next to a neutral expression). To validate the stimuli used in experiment 1 and to further explore humans' perception of chimpanzee emotional expressions, we conducted a third experiment (experiment 3) in which humans viewed the chimpanzee stimuli from experiment 1.

Based on the evidence of emotion biases reported for both species (e.g. chimpanzees (19), humans (45)) and evolutionary relevance of fear/threat-related stimuli (46), we expected that when chimpanzees and humans view neutral nonsocial *fish tank* primers (i.e. videos of fish swimming in a colorful aquarium), they exhibit an unaffected, natural emotion bias by prioritizing images showing emotional over neutral expressions in their initial orientation (i.e. first look) as well as sustained attention (i.e. looking time), especially toward images showing fearful (over playful) expressions (*prediction 1*-basic emotion bias). When compared to *resting* primers (i.e. two individuals sitting or walking calmly), these emotion biases may be attenuated, as subjects' attention may be more drawn toward the *neutral* facial expressions congruent with the *resting* primers, whereas *fish tank* primers have no link to any specific facial expression.

Presuming that both chimpanzees and humans are affected by the bodily primers, we expected them to exhibit differences in looking behavior toward congruent and incongruent facial images. Recent research using anticipatory looking measures in humans has revealed that attention is particularly devoted to expected events related to specific priming stimuli (44, 47). Following this, our next prediction was that attention (i.e. both initially and sustained) should be prioritized toward images of facial expressions *congruent* rather than incongruent with a previous bodily primer. Thus, following *fear* primers, we predicted attention to be steered toward images of *threat-related* facial expressions over those including *play face/smiling* expressions (*prediction 2*-*fear* priming); on the contrary, following *play* primers, we predicted attention to be more strongly driven toward images showing *play face/smiling* expressions over those containing *threat-related* expressions (*prediction 3*-*play* priming).

## Methods

### Study sites and participants

#### Experiment 1: chimpanzees viewing chimpanzee stimuli

Research with chimpanzees was carried out at Basel Zoo, Switzerland, from November 2020 to May 2021. For information on the study site and group composition, see Text S1 and Table S1. The chimpanzees were habituated to computerized experiments and had participated in eye-tracking research before this study. The experiment initially involved eight participants (Table S1); however, one did not pass the calibration, and another never completed the initial attention validation test (details below), yielding a final sample of six chimpanzee subjects (four females, age mean = 15 years, SD = 11 years).

#### Experiment 2: humans viewing human stimuli

Given the practical difficulties in estimating sample size for a mixed model with nested structure, we predetermined sample size based on a power analysis using G*Power Version 3.1 (48) for a repeated measures ANOVA to detect a medium effect of *f* = 0.25; with a statistical power of 0.80 and a significance level of 0.05; effect size was taken from a prior study suggesting at least a medium effect of the cross-modal perceptual integration of emotional expressions (49). Based on this analysis, we targeted to collect 24 participants. In total, we recruited 27 participants (all undergraduate students; 19 women; age mean = 20.07 years, SD = 1.96 years), of which three participants were excluded due to technical problems with the eye-tracker. The experiment was conducted in a laboratory room at Leiden University, in the Netherlands, from April to May 2022. Inclusion criteria were no prior history of clinically diagnosed psychiatric conditions and normal/corrected-to-normal vision with contact lenses. Participants were recruited through the Leiden University research participation management tool (SONA) and compensated with course credits.

#### Experiment 3: humans viewing chimpanzee stimuli

The research was conducted with 25 undergraduate students (20 women; age mean = 20.28 years, SD = 2.49 years) in a laboratory room at Leiden University, from April to May 2022. One participant was excluded from the data analysis, as they were unable to complete the experiment due to technical problems with the eye-tracker. None of these participants had participated in experiment 2. All other details were identical to experiment 2. Although we did not run a formal survey to enquire about participants' previous experience in observing chimpanzees, the psychology undergraduate students we sampled were unlikely to have had extensive exposure to great apes (beyond what a standard member of the public would have) nor received training in great ape communication research; they thus did not represent an expert sample that could potentially yield improved evaluation of chimpanzee bodily or facial emotion expressions (50).

### Research apparatus

#### Experiment 1: chimpanzees viewing chimpanzee stimuli

The eye-tracking apparatus consisted of a TX300 Tobii Pro screen-based eye-tracker and a 23″ LCD (Liquid Crystal Display) monitor (screen resolution of 1,920 × 1,080 pixels; aspect ratio 16:9). The eye-tracker had a vertical sync frequency of 49–75 Hz and a horizontal sync frequency of 54.2–83.8 kHz with a response time of 5 ms. A Microsoft HD LifeCam Cinema webcam was installed on top of the eye-tracker to film the trial. In terms of the gaze filter, we used the fixation filter in Tobii Pro Lab (version 1.138) to distinguish saccades from fixations. Chimpanzees sat or stood in front of the screen at an average distance of 61.9 cm (SD = 1.3 cm) and watched the screen while drinking juice through a drinking nozzle (for details, see Text S2). Gaze behavior was monitored through an extended monitor placed in an adjacent room by the experimenter separate from the chimpanzees' cage using the Tobii built-in Live Viewer function.

#### Experiment 2: humans viewing human stimuli

We used a Tobii T120 eye-tracker mounted on a 17″ TFT (thin-film transistor) monitor with a screen resolution of 1,280 × 1,024 pixels (aspect ratio 5:4), which had a vertical sync frequency of 56–75 Hz and a horizontal sync frequency of 30–60 kHz and a response time of 4 ms. The fixation filter in Tobii Studio (version 3.4.8) was used to distinguish saccades from fixations. The experiment took place in a sound-isolated laboratory room with ambient light. Participants sat at a table with a chinrest to minimize head movements and viewed the screen at a 60 cm distance. Participant gaze behavior was monitored in an extended monitor placed in an adjacent room by the experimenter using the Tobii built-in Live Viewer function.

#### Experiment 3: humans viewing chimpanzee stimuli

The setup was identical to experiment 2.

### Experimental design and stimuli

For both species, the experiment comprised a four (primer conditions: *fish tank*, *resting*, *play, fear*) × two (image pairs: *play face*/*smiling* vs. *neutral expression, fear/bared-teeth* vs. *neutral expression*) within-subject design. Each individual was exposed to 160 trials, administered in 40 sessions (i.e. four trials per session, with all of the four conditions represented in a single session). The order of the primer conditions within each session was randomized.

#### Experiment 1: chimpanzees viewing chimpanzee stimuli

Stimuli contained videos of bodily expressions (i.e. primers) and images of facial expressions of chimpanzees in wild or zoo settings, which were kindly shared by other primatologists (see Acknowledgments). We included only stimuli of unfamiliar chimpanzees to avoid variation in perception due to rank differences and personal experiences between participating subjects and individuals depicted in the stimuli. There were 20 unique videos for each primer (*fish tank*, *resting*, *fear*, and *play*), each 3 s in length. *Play* primers depicted two individuals who were either engaged in bodily contact, e.g. by tickling or wrestling each other, or chasing after each other in a playful manner (e.g. Movie S1). *Fear* primers showed two chimpanzees engaged in conflict, with tense bodily movements, chasing, and/or physically attacking each other (e.g. Movie S2). *Neutral* control primers contained scenes of social *resting* or *fish tank*; *resting* primers showed two chimpanzees either sitting, calmly moving, standing, or lying close to one another without actively engaging in social activity (e.g. Movie S3). *Fish tank* primers showed real fish of different colors and sizes swimming calmly in a colorful aquarium (e.g. Movie S4). Primers were selected based on video quality and clarity of the corresponding social context. The videos were counterbalanced as much as possible for the age and sex of chimpanzee actors (Table S2). Facial expressions and vocalizations were masked from primer videos in Adobe Premier Pro CC 2020 (version 14.3). Further details on video content and preparation can be found in Texts S3 and S4.

**Fig. 1.**
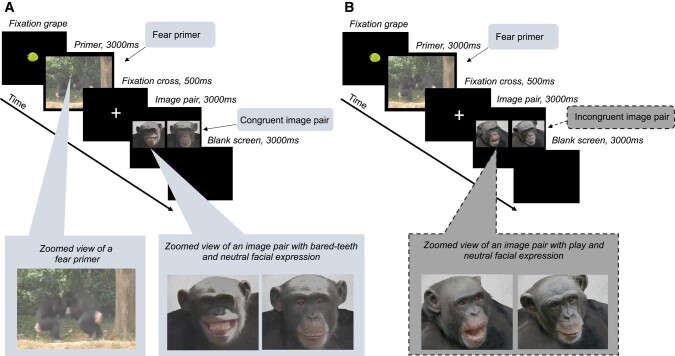
Example of two eye-tracking trials for chimpanzees for the *fear* primer condition. A) Congruent trial (i.e. image pair with emotionally congruent facial expression next to a neutral face). B) Incongruent trial (i.e. image pair with emotionally incongruent facial expression next to a neutral face). See for other stimuli examples and original gaze patterns Movies S1–S4 (chimpanzees) and Movie S6 (humans). The images were kindly shared by other primate experts, listed and credited in our acknowledgments.

Each primer was matched with two corresponding image pairs, which ideally showed the same individuals as in the video, or if not possible, chimpanzees of a similar age and sex class. One image pair contained a neutral facial expression adjacent to a facial expression congruent with the primer (e.g. *play face* following *play* primer) and another image pair showed a neutral facial expression next to a facial expression incongruent with the primer (e.g. *bared-teeth* expression following *play* primer; see Fig. 1). One trial consisted of a primer and a subsequently presented image pair. See further details on image pair content and preparation in Texts S3 and S5.

Given that bared-teeth expressions can have different communicative meanings depending on whether they occur silently or voiced (51), we conducted a test to verify whether variation in expression intensity could affect attention levels. As the static images of bared-teeth expressions had been provided by other chimpanzee experts before this study, it cannot be ascertained with certainty post hoc whether these expressions were voiced or not. To nonetheless address this issue, we classified expressions into “low intensity” (bared teeth with clenched teeth, mouth slightly open or closed but lips withdrawn, mouth corners retracted such that teeth are exposed) and “high intensity” (bared teeth with mouth open, lips strongly withdrawn, complete exposure of teeth). The analysis was based on a reduced dataset (i.e. only bared-teeth expressions as static images) and revealed that variation in bared-teeth intensity had no evident impact on attention, confirmed by model outputs as well as leave-one-out cross-validations (LOOIC) of full and reduced models (52) (Table S8).

In sum, 80 unique image pairs (40 image pairs with *bared-teeth* expressions vs. *neutral* expressions and 40 image pairs with *play faces* vs. *neutral* expression) were matched with 40 distinct *emotional* primers (20 *fear*; 20 *play*). Each primer was matched once with an image pair with a *congruent* emotion image and once with an image pair with an *incongruent* emotion image, presented in random order. In the second step, the same 80 image pairs were assigned to 40 neutral *control* primers (20 *resting* and 20 *fish tank*). For the *resting* primer, we matched image pairs according to similar identities, physical properties, age, and sex of the respective scene.

The number of first and repeated views of image pairs after an *emotional* or *control* primer was randomized across sessions while ensuring that one-half of trials contained first views of image pairs following *emotional* primers and the other half of first views of image pairs following *neutral* control primers. When image pairs were presented a second time, the positions (left/right) of the emotional face and neutral face were flipped. The position of the emotion image was likewise randomized within the session and counterbalanced, such that an equal number of emotion images was shown on the left and right sides.

To verify that chimpanzee attentional patterns were driven by the content rather than the physical properties of the stimuli, we instigated an attention validation test (53). To this end, we prepared single images of food items against a white background, which reflect the groups' typical dietary regime in captivity (vegetables, fruits, nuts, eggs). The images were presented in image pairs, once in their original form (*nonscrambled*) and once disrupted in their configuration by a scrambling of pixels (*scrambled*). *Scrambled* images were presented either on the left or right side of the corresponding *nonscrambled* image, a position that was randomized within the session and counterbalanced (i.e. an equal number of left/right images appeared within each session). One session contained four trials (i.e. four image pairs), yielding a total of 24 trials and 6 sessions. Details on and examples of scrambled images are found in Text S5 and Movie S5.

#### Experiment 2: humans viewing human stimuli

For the primers, we initially prepared 90 videos of spontaneous (nonposed) human social interactions. This included 30 *fear* primers, in which individuals are victims of scary pranks; 30 *play* primers, where two or more people laughed together; and 30 neutral *resting* primers, including scenes where people calmly sat or walked. The scenes were selected from YouTube and free stock videos on the internet. The videos were selected based on the highest visibility and intensity of the main actors displaying emotions (see Movie S6 as an example). Using Adobe Premier Pro, the videos were cut to 3 s in length and facial expressions and sounds were masked. The 90 edited video scenes were then rated for emotion category, valence, and intensity of the bodily expression, as well as the authenticity of the actors depicted in the scene, by 5 researchers specialized in human emotion communication. Based on this rating, the top 20 videos for each primer condition were selected as stimuli, yielding 40 emotional primers, including 20 *fear* and 20 *play*, and 20 *resting* primers. Details on video contents and preparation can be found in Text S4 and stimuli ratings in Table S3. As an additional control primer, we used the same 20 *fish tank* scenes as in experiment 1.

For images showing facial expressions, we selected 80 unique image pairs (40 containing *fearful* faces vs. *neutral* expressions and 40 containing open-mouth *smiling* faces vs. *neutral* expressions) from the Chicago Face Database (54) and Radboud Face Database on posed expressions (55). For further information regarding face image content and preparation, see Text S5 and Movie S6. Each primer was matched once with an image pair with a *congruent* emotion image and once with an image pair with an *incongruent* emotion image, presented in random order. As the actors in the images were not identical to the people shown in the primers, we matched their gender and physical appearance as much as possible (i.e. see Table S4 for an overview of gender combinations). The randomization and counterbalancing of emotion image positions in image pairs were identical to experiment 1.

#### Experiment 3: humans viewing chimpanzee stimuli

The experimental design and stimuli were identical to those used in experiment 1; the testing conditions were as described in experiment 2.

### Procedure

#### Experiment 1: chimpanzees viewing chimpanzee stimuli

Before testing started, each subject's eye gaze was calibrated using the inbuilt two-point calibration option in Tobii Pro Lab, a procedure also used in other studies using eye-tracking in primates (14). The calibration results are of excellent quality for current eye-tracking standards (see Text S6).

Next, we administered the attention validation test to investigate looking time toward *scrambled* versus *nonscrambled* images of food items. All six sessions could be administered in one day if the subject was motivated. Each trial started with a black fixation screen showing a green grape (95 mm × 85 mm) in a central position. Once the subject fixated on the grape, the experimenter manually initiated the automatic run of the trial, which included the presentation of an image pair for 3 s. During the entire test, the experimenter monitored the participant gaze by looking at the Tobii Pro Lab Live view output; if the participant looked away for >2 s during the presentation of the image pair, the trial was coded as “missed” and repeated the next day. We found that subjects looked at the original images substantially longer than *scrambled* images (see Text S7 and Table S6.1), suggesting that stimuli content, not low-level physical features like color and luminance, guided their attention.

Once subjects had completed the attention validation test, they progressed to the main experiment. To avoid loss of concentration, each subject could complete maximally three sessions per day. Before the first session on a given day, their gaze was validated by manually checking gaze fixations on at least two points of a four-point grid using the inbuilt validation function in Tobii Pro Lab. In addition, attention levels were verified in a warm-up trial with an identical structure of subsequent experiment trials: a fixation grape on a black screen, 3 s video of a solitary, nondangerous animal (horse, donkey, zebra, cow, or deer) followed by a 3 s image pair showing two adjacent images of the corresponding animals' neutral face. Only if attention was deemed sufficient, subjects progressed to the main experiment.

Here, each trial started with a black fixation screen showing a green grape (95 mm × 85 mm) in the central position (Fig. 1). Once the participant's gaze was fixated on the grape, the experimenter manually started the test. Once initiated, the trials ran automatically. Each trial included a 3,000 ms primer, a consecutive 500 ms fixation cross to centralize gaze, a subsequent 3,000 ms image pair, and a final 3,000 ms black screen (Fig. 1). During the test, attention was manually monitored by the experimenter; if the subject looked away for >2 s, the trial was repeated on another day. Since chimpanzees' attention span is generally more restricted compared to that of humans, we played each trial individually (i.e. instead of instigating an automatic run of all four trials within a session, like in experiment 2, we manually started each trial once subjects fixated on the initial fixation grape, see Movies S1–S4). There were maximally four repetitions of a trial; if the trial was not completed at the fourth attempt, it was discarded. If the subject remained seated at the apparatus after having completed their maximum number of daily sessions, a blue screen was shown until the subject left.

#### Experiment 2: humans viewing human stimuli

Following informed consent, the participant was led into the testing room. They sat comfortably in front of the monitor and were instructed to minimize any head movements. If necessary, the height of the chinrest was adjusted. The participant first underwent the built-in Tobii Studio nine-point calibration. If necessary, the calibration was repeated until maximum accuracy was achieved. Additionally, the experimenter validated the calibration accuracy at the beginning of the first session by visually checking the participant's fixations on small pictures of a 3 × 3 grid on a black background; the validation continued until at least two points were fixated (56). After the validation, the participant was instructed to freely view the videos and images on the screen.

Each test trial started with a small fixation grape (95 mm × 85 mm) at the center of black background for 1,000 ms to centralize the participant's attention. Then, the participant viewed the 3,000 ms primer, a consecutive 500 ms fixation cross to centralize gaze, a subsequent 3,000 ms image pair, and a 3,000 ms final blank black screen, identical to Experiment 1. After every tenth session, the participant took a short break (2–3 min) between trials. After completing the eye-tracking task, the participant was given a short Qualtrics survey to state their age and gender and was debriefed about the aim of the study.

#### Experiment 3: human viewing chimpanzee stimuli

The design and procedure were identical to experiment 2, except that the participants viewed the chimpanzee stimuli of experiment 1.

### Data analysis (experiments 1–3)

For image pairs, rectangular areas of interest (AOIs) were drawn on the emotional and neutral image, with a 20% margin to accommodate calibration error (57). We extracted the latency to first fixation on the AOIs to create the variable of *initial orientation*, and total fixation duration on the AOIs to generate the variable of *sustained attention*. We excluded trials in which latency to first fixation on either of the AOIs was faster than 150 ms, to exclude any first fixation data points that occurred independent of stimulus perception (58). For *initial orientation*, the time to first fixation on the emotional image was later converted to 1 (faster first fixation on the emotional over neutral image) or 0 (slower first fixation on the emotional image over neutral image) for each trial; this binary variable is referred to as *TFF_EmoBinary_*. Notably, a *TFF_EmoBinary_* >0.5 represents an *emotion bias* in initial orientation, meaning that participants fixated faster on emotional images compared to neutral images.

For *sustained attention*, we computed a proportion of looking time toward the emotional image, referred to as *TFD_EmoProp_*. This was done by calculating the total fixation duration on the emotional image divided by the total fixation duration on both images, i.e. the emotional and neutral image. A *TFD_EmoProp_* >0.5 thus likewise represents an *emotion bias*, insofar as the participant looked longer at the emotional image compared to the neutral image. As *TFD_EmoProp_* was a proportional value, which did not consider the variance in participants' overall looking time, we additionally created a *Weight* variable for each trial. *Weight* was calculated for each individual by dividing the total looking time toward the two AOIs in each trial by the average looking time on the two AOIs across all trials.

#### Statistical analysis

To test our predictions, we fitted Bayesian generalized mixed models using the Stan computational framework (http://mc-stan.org/) using *brms* (59) in R (version 4.1.1). Each model included four Markov chain Monte Carlo (MCMC) chains, with 4,000 iterations per chain, including 1,000 warm-up iterations. We checked model diagnostics via model summaries and the function *launch_shinystan().* We found an accurate reflection of the original response values by the posterior distributions, acceptable R-hat statistics <1.05, sufficient effective samples >1,000, and no divergent transitions in MCMC chains. We used weakly informed priors to penalize extreme parameter values. This included a prior with a normal distribution, mean = 0 and a scale parameter of 1 for the *intercept* as well as *b* estimates, and a prior with a half *cauchy* distribution, mean = 0 and scale parameter of 1 for *SD*. Following the Bayesian Analysis Reporting Guidelines (BARG) (60), we report the *median* estimate to describe the central tendency, the median absolute deviation (*MAD*) and 89% credible intervals (*CrIs*) related to the limits of highest density intervals (*HDIs*) of the estimates. We chose 89% over the arbitrary convention of 95% as recommended by McElreath (61). Note that, for *robust* interaction terms (i.e. where the 89% *CrIs* do not overlap with zero), we further contrasted results on attention across priming conditions and image pairs using the package *emmeans* (62). We additionally computed the probability of direction (*pd*) ranging from 50 to 100% as an index of effect existence using *bayestestR* (63). Emotion biases were also determined using *emmeans* and confirmed if point estimates were >0.5 and 89% *CrIs* not crossing 0.5.

#### Model specificities for experiment 1

For the attention validation model, we fitted a *zero-inflated beta* distribution. This enabled us to model the probability of 0 and 1 (trials with fixations on one image over the other) to assess attention on *nonscrambled* images compared to *scrambled* images. For the main experiment, we fitted two Bayesian mixed models; model 1 included initial orientation (*TFF_EmoBinary_*) as the response variable, fitted with a *Bernoulli* distribution; model 2 included sustained attentions (*TFD_EmoProp_*) as the response variable, fitted with a *zero-one-inflated beta* distribution. Both models included as independent variables trial repetition (*no/yes)* and an interaction between primer conditions and image pairs. Moreover, primer ID and image pair ID, as well as sessions nested within-subject ID, were included as random intercepts. As our chimpanzee subjects varied in age (range 4–43 years) and sex (five females, one male), we ran LOOIC comparisons to verify whether age and sex improves model accuracies, thus are worth being added as covariates to our models. As there were no improvements in model accuracies when adding age or sex as covariates to our existing models 1 and 2 (Table S8), we present the parsimonious (reduced) models in our main paper as recommended for Bayesian modeling (Ref. (64)).

#### Model specificities for experiment 2

Model 3 was fitted with initial orientation as the response variable and model 4 with sustained attention as the response variable; the fitted distributions and independent variables were the same as in experiment 1, except that we did not include the trial repetition, as there was no repeated trial for humans. We also included participant gender as covariate to account for potential gender variation.

#### Model specificities for experiment 3

Model 5 had initial orientation and model 6 had sustained attention as its response variable; fitted distributions, independent variables and random effects were identical to experiment 1 (except trial repetition, which was not applied in this case).

## Results

We predicted basic emotion biases in the *fish tank* priming condition, especially toward *fearful* facial expressions (*prediction 1*, Table 1). We further predicted congruency effects related to bodily emotion *priming* conditions, insofar as participants look faster (i.e. initial orientation: *TFF_EmoBinary_*) and longer (i.e. sustained attention: *TFD_EmoProp_*) toward emotion images whose facial expressions correspond to the perceived valence of the emotion primer (*predictions 2 and 3*, Table 1). For descriptive results on average looking times across groups, see Text S8.

**Table 1.** Summary of results in relation to predictions.

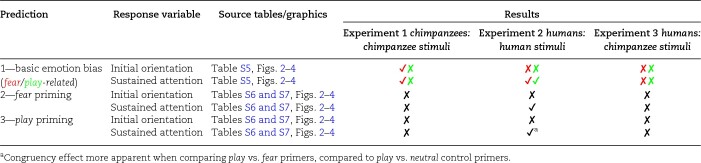

### Experiment 1: chimpanzees viewing chimpanzee stimuli

#### Initial orientation (TFF_EmoBinary_)

**Fig. 2.**
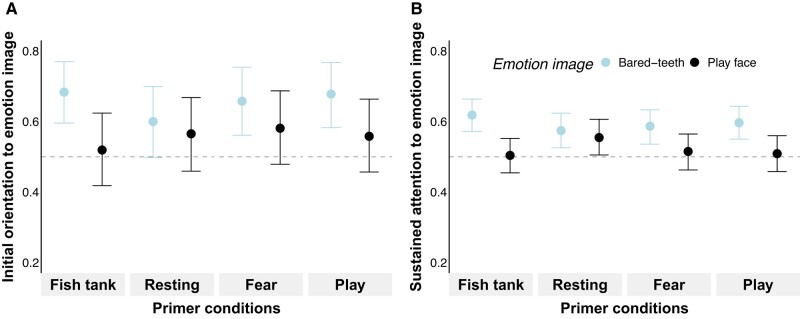
Results of experiment 1 (*chimpanzees viewing chimpanzee stimuli*) with initial orientation to emotion image (*TFF_EmoBinary_*: A) and sustained attention to emotion image (*TFD_EmoProp_*: B) depending on emotion image and primer condition. Values >0.5 denote emotion biases toward emotional images compared to neutral images. Points indicate estimated posterior medians and hinges indicate 89% *CrIs* based on results using *emmeans* (see Table S7.1).

In line with *prediction 1*, we found a basic emotion bias, which was mainly *fear-related* (Table S5.1, Fig. 2A). This meant that the chimpanzees' bias in initial orientation toward images depicting *bared-teeth* expressions over neutral faces was stronger compared to the bias in initial orientation toward *play faces* over neutral faces (Δestimate*_Fish:Baredteeth-Fish:Playface_* = 0.69 ± 0.31, 89% *CrI* [0.21, 1.19], *pd* = 98.90%; Table S7.1 model 1). Contrary to *predictions 2 and 3* though, this emotion bias was not affected by emotional primers; there was no interaction between primers and images (Fig. 2A and Table S6.2 model 1), indicating no congruency effects in chimpanzees. We further found no evidence for habituation effects of repeated trials (Table S6.2 model 1).

#### Sustained attention (TFD_EmoProp_)

In line with *prediction 1*, we found a basic emotion bias also for sustained attention, again being mainly *fear-related* (Table S5.1 and Fig. 2B). This meant that the chimpanzees' bias in sustained attention toward *bared-teeth* expressions over *neutral* faces was stronger compared to the bias in sustained attention toward *play face* expressions over *neutral* faces (Δestimate*_Fish:Baredteeth-Fish:Playface_* = 0.46 ± 0.14, 89% *CrI* [0.25, 0.69], *pd* = 99.95%; Table S7.1 model 2). Moreover, we found an interaction between the primer conditions and images (Fig. 2B and Table S6.2 model 2). To further investigate whether the direction of the interaction was in line with *predictions 2* and *3*, we contrasted the results on *TFD_EmoProp_* for emotional images across primers using *emmeans*. We found no evidence for the expected congruency effects related to emotional primers (Table 1). Instead, there was a persistent *fear-related* emotion bias, except for the *resting* primer, where the *fear-related* emotion was attenuated (Δestimate*_Resting:Baredteeth-Resting:Playface_* = 0.08 ± 0.15, 89% *CrI* [−0.16, 0.31], *pd* = 70.76%; Table S7.1 model 2). The lacking congruency effects were further confirmed by no evident increases of *TFD_EmoProp_* toward images of *bared-teeth* expressions following *fear* primers compared to *fish tank* primers (Δestimate*_Fish:Baredteeth-Fear:Baredteeth_* = 0.13 ± 0.14, 89% *CrI* [−0.09, 0.35], *pd* = 82.88%; Table S7.1 model 2). We found no evidence for habituation effects of repeated trials (Table S6.2 model 2).

### Experiment 2: humans viewing human stimuli

#### Initial orientation (TFF_EmoBinary_)

**Fig. 3.**
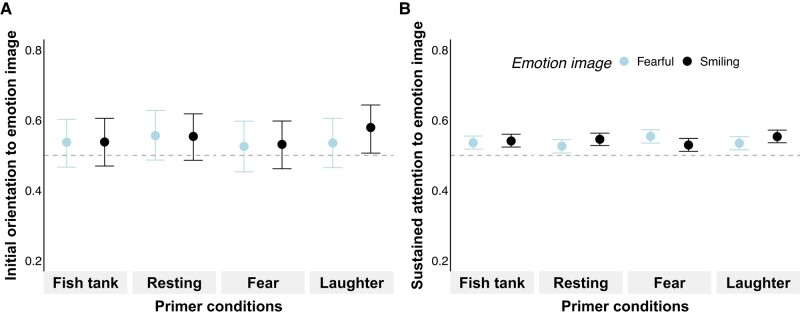
Results of experiment 2 (*humans viewing human stimuli*) with initial orientation to emotion image (*TFF_EmoBinary_*: A) and sustained attention to emotion image (*TFD_EmoProp_*: B) depending on emotion image and primer condition. Values >0.5 denote emotion biases toward emotional images compared to neutral images. Points indicate estimated posterior medians and hinges indicate 89% *CrIs* based on results using *emmeans* (see Table S7.2).

In contrast to *prediction 1*, we found no evidence of basic emotion biases in humans' initial orientation (Table S5.2 and Fig. 3A). There was also no evidence of an interaction between primer conditions and images in terms of humans' initial orientation (*predictions 2* and *3*, Fig. 3A and Table S6.3 model 3), and no evidence of a gender effect (Table S6.3 model 3).

#### Sustained attention (TFD_EmoProp_)

In line with *prediction 1*, we found basic emotion biases toward *fearful* and *smiling* facial expressions over *neutral* ones (Table S5.2 and Fig. 3B), with no difference in the bias between *fearful* and *smiling* expressions (Δestimate*_Fish:Fearful-Fish:Smiling_* = −0.02 ± 0.05, 89% *CrI* [−0.09, 0.06], *pd* = 65.92%; Table S7.2 model 4). Moreover, we found a robust interaction between primer conditions and images (Fig. 3B and Table S6.3 model 4). To investigate whether the direction of the effects was in line with *predictions 2* and *3*, we contrasted *TFD_EmoProp_* for emotion images across primer conditions using *emmeans*. As expected, humans exhibited a *fear-related* emotion bias, i.e. stronger *TFD_EmoProp_* toward images of *fearful* compared to *smiling* facial expressions, following *fear* primers (Δestimate*_Fear:Fearful-Fear:Smiling_* = 0.10 ± 0.05, 89% *CrI* [0.02, 0.17], *pd* = 98.22%; Fig. 3B and Table S7.2 model 4). To further validate whether this *fear-related* emotion bias was facilitated by *fear* primers, we contrasted the respective outcomes with the two *neutral* control primers (i.e. *fish tank* and *resting*). As expected, the *fear-related* emotion bias was particularly enhanced after viewing the *fear* primer compared to the *resting* primer (Δestimate*_Fear:Fearful-Resting:Fearful_* = 0.11 ± 0.05, 89% *CrI* [0.03, 0.19], *pd* = 98.76%; Fig 3B and Table S7.2 model 4). Although the effect was less robust, a similar pattern also emerged when comparing *fear* and *fish tank* primers (Δestimate*_Fish:Fearful-Fear:Fearful_* = −0.07 ± 0.05, 89% *CrI* [−0.15, 0.01], *pd* = 92.54%; Fig. 3B and Table S7.2 model 4). There was also weak evidence for an equivalent *play-related* emotion bias following *play* primers (Δestimate*_Play:Fearful-Play:Smiling_* = −0.08 ± 0.05, 89% *CrI* [−0.15, 0.00], *pd* = 94.05%; Fig. 3B and Table S7.2 model 4) and when comparing the *play* primer with the two *neutral* control primers (Table S7.2 model 4). Additionally, there was an enhanced *play-related* emotion bias following *play* primers compared to *fear* primers (Δestimate*_Fear:Smiling-Play:Smiling_* = −0.10 ± 0.05, 89% *CrI* [−0.17, −0.02], *pd* = 97.65%; Fig. 3B and Table S7.2 model 4). We found no evidence for a gender effect (Table S6.3 model 4).

### Experiment 3: humans viewing chimpanzee stimuli

#### Initial orientation (*TFF_EmoBinary_*)

**Fig. 4.**
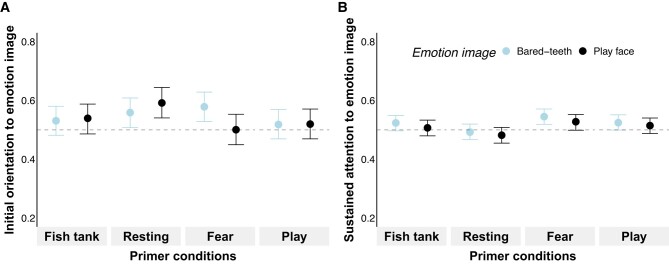
Results of experiment 3 (*humans viewing chimpanzee stimuli*) with initial orientation to emotion image (*TFF_EmoBinary_*: A) and sustained attention to emotion image (*TFD_EmoProp_*: B) depending on emotion image and primer condition. Values >0.5 denote emotion biases toward emotional images compared to neutral images. Points indicate estimated posterior medians and hinges indicate 89% *CrIs* based on results using *emmeans* (see Table S7.3).

Contrary to *prediction 1*, we found no evidence of clear basic emotion bias when humans viewed chimpanzee stimuli (Table S5.3 and Fig. 4A). There was however an interaction between the primer conditions and images (Fig. 4A and Table S6.4 model 5). To further investigate whether the effects were in line with *predictions 2* and *3*, we contrasted *TFF_EmoBinary_* for emotion images across primer conditions and images using *emmeans*. We found that humans' initial orientation toward chimpanzee *bared-teeth* expressions (compared to *play faces*) was strongly enhanced by *fear* primers (Δestimate*_Fear:Baredteeth-Fear:Playface_* = 0.31 ± 0.14, 89% *CrI* [0.10, 0.55], *pd* = 98.70%; Fig. 4A and Table S7.3 model 5). Yet, when we compared this *fear-related* emotion bias between *fear* primers and the relevant *neutral* control primers (i.e. *fish tank* and *resting*), we found no evidence that this effect was specifically caused by *fear* primers (Fig. 4A and Table S7.3 model 5).

Likewise, the chimpanzee *play* primer did not seem to influence humans' initial orientation, as there were no clear increases of *TFF_EmoBinary_* toward *play faces* compared to *bared-teeth* expressions (Δestimate*_Play:Baredteeth-Play:Playface_* = −0.01 ± 0.14, 89% *CrI* [−0.22, 0.22], *pd* = 52.37%; Fig. 4A and Table S7.3 model 5). Interestingly though, in contrast to the predicted congruency patterns, humans' emotion bias toward chimpanzee *play faces* (compared to *neutral* faces) was not particularly enhanced by *play* but rather by *resting* primers (Δestimate*_Play:Playface-Resting:Playface_* = −0.29 ± 0.17, 89% *CrI* [−0.57, −0.02], *pd* = 95.52%; Fig. 4A and Table S7.3 model 5). There was no evidence of a gender effect (Table S6.4 model 5).

#### Sustained attention (*TFD_EmoProp_*)

In terms of *prediction 1*, there was no robust emotion bias toward images of chimpanzee facial emotion expressions in human participants (Fig. 4B and Table S5.3; Δestimate*_Fish:Baredteeth-Fish:Playface_* = 0.07 ± 0.06, 89% *CrI* [−0.03, 0.16], *pd* = 87.07%; Table S7.3 model 6). There was also no evidence of interaction effects between primer conditions and images, thus no support for *predictions 2* and *3* (Fig. 4B and Table S6.4 model 6) and no gender effect (Table S6.4 model 6).

## Discussion

This study investigated how chimpanzees and humans process emotions from bodily movements and whether they associate bodily cues with corresponding facial expressions. Although chimpanzees exhibited a strong preference for fearful emotion expressions over play faces and neutral faces across bodily primers (except after *resting* primers; Table 1, *prediction 1*), this bias was unexpectedly not contingent on the congruency of the bodily primer. By comparison, humans exhibited an emotion bias toward both fearful and smiling faces over neutral faces in sustained attention, suggesting an equally relevant role of positive and negative emotions in visual communication. Importantly, unlike in chimpanzees, the attentional preference toward images of facial expressions in humans was contingent on the congruency of the bodily primer (*prediction 2*): when humans viewed *fear* primers, they subsequently looked longer toward *fearful* facial expressions as compared to when viewing neutral *resting* primers. Moreover, in line with our third prediction (*prediction 3*), when viewing *play* primers, humans looked longer toward images of *smiling* facial expressions compared to when viewing the *fear* primers. When humans viewed chimpanzee stimuli, we found no evidence of a clear emotion bias, nor evidence of relevant priming effects (Table 1); instead of the expected priming effect, humans looked faster toward images of chimpanzees' *play face* expressions when viewing chimpanzee *resting* primers compared to chimpanzee *play* primers. These results suggest difficulty in the ability of humans to infer emotions from chimpanzee signals.

What could explain the predominant *fear-related* bias in chimpanzees and a relatively attenuated respective bias in humans? Although several studies have demonstrated *fear-related* emotion biases in humans (38, 41, 45), there are likewise studies showing opposite or even null effects, suggesting that emotion biases can vary according to experimental paradigms, the stimuli being used as well as participants' age (i.e. although children exhibit positivity biases in face processing, some studies show a reversed effect even toward negativity biases over the course of development, see for review Ref. (65)). This variability in emotion biases across human studies, including our own, suggests that humans may not exhibit clear prioritization for specific valence types and that contextual priming (or other factors) may affect emotion processing. In contrast, chimpanzees appear to engage in a relatively biased form of emotion perception (i.e. toward *fear-related* facial expressions) that is comparatively less impacted by bodily interaction cues. However, given the lack of an emotion bias of chimpanzees in other relevant studies (20), this conclusion is still debatable; more research on emotional perception in different chimpanzee populations, using diverse experimental paradigms, is required to provide clearer answers under what circumstances this species exhibits (*fear-related*) emotion biases.

In terms of bodily priming, we found relevant congruency effects in humans but not in chimpanzees (*predictions 2* and *3*). Unlike chimpanzees, humans exhibited an attentional bias toward images of facial expressions congruent with the preceding emotional primer. A mild exception to this pattern was when comparing *play* with neutral *fish tank* and *resting* primers; here, the effects of priming were relatively attenuated (yet still evident) compared to when comparing *fear* with neutral *fish tank* and *resting* primers. One might argue that humans considered the presumably “neutral” control stimuli to be slightly pleasant, and thus more similar to the *play* primer. This explanation matches the consensus that affiliative states are more ambiguous compared to fear-related states, with greater adaptive value of the latter; misunderstanding threatening situations could imply risk of survival (see also Ref. (45) for evidence on stronger cross-cultural variation in perception related to affiliative emotional states compared to fear/anger-related ones). However, it is noteworthy that in our study, our expert raters converged on clear valence ratings of human stimuli, with *play* primers being rated highly positive and the *control* primers as clearly neutral (Table S3). Our results could nonetheless be interpreted insofar as *fear-related* primers elicit stronger priming responses compared to *affiliative* or *neutral* ones, possibly due to greater adaptive value of the former (66).

But why did humans exhibit bodily priming effects and chimpanzees not? Research has demonstrated that humans can broadly discriminate “positive” and “negative” emotional states in terms of isolated bodily cues, yet not necessarily based on isolated facial expressions (67). Furthermore, when facial expressions are presented *alongside* bodily information, peoples' valence ratings of specific scenes improve (67). It is thus possible that in humans, compared to chimpanzees, facial expressions may be less tethered to the underlying emotional states and more variably affected by bodily cues. Indeed, evidence from field studies in naturalistic settings points to ambiguous meanings and functions of facial movements in humans, rather than facial signals being read-outs of emotional states (68). Evidence of cultural variation in emotional expressions of humans also supports this view, insofar as emotional facial movements can be affected by cultural processes (69, 70) and subject of voluntary control (Ref. (71), but see Refs. (72, 73)).

In chimpanzees, by contrast, facial expressions appear to have clear ties to a specific context. While human laughter and smiles can be linked to range of social situations, including anxiety, embarrassment, politeness, and amusement (74), chimpanzee laughter and play faces (i.e. aka open-mouth faces) are emitted almost exclusively in playful settings (Ref. (75), see for a detailed review in Ref. (76)). Moreover, although chimpanzees use the bared-teeth expression, a morphological homologue of the human smile (Ref. (77), but see Ref. (76)), across multiple contexts, this expression nonetheless appears to be most frequently deployed in submissive contexts, as a potential signal of affinity (51, 75). Not displaying benign signals as such might incur risks of severe aggression in a community highly affected by dominance hierarchies (78). For these reasons, our subjects' fear-related emotion bias toward the highly relevant bared-teeth expressions may have superseded any priming effects.

Interestingly, our lack of evidence of emotional priming in chimpanzees contrasts findings from a former match-to-sample study, in which chimpanzees matched congruent facial expressions with corresponding contextual scenes above chance (25). It is to note however that this discrepancy could be related to differences in the methodologies and stimuli used, as well as individual differences. Match-to-sample requires substantial training, which eliminates spontaneity of emotional perception. Moreover, the scenes used in the match-to-sample study (25) were not related to naturalistic bodily movements of conspecifics, but were scenes relevant to life in captivity, spanning fear and anxiety (e.g. veterinarians, injections, and medical procedures) and reward/play (e.g. toys and bottles of juice). Nonetheless, given that chimpanzees in principle can associate facial and contextual cues, what else could explain a respective lack of priming effects in our study?

First, it may be that the bodily behavior in play and conflict aggression, as used in our study, are hard to distinguish if no other clues are provided. Researchers often highlight that play-fighting resembles aggression in many ways, including close-contact, rough, and fast-paced bodily movements (79–81). Evidence supporting this view is that the chimpanzee scenes, when shown to human participants, did not elicit differential attentional patterns toward consecutive facial expressions, suggesting also humans may have had difficulties in discerning playful and conflictual scenes. The play face may act as crucial communicative signal to emphasize benign intentions in chimpanzee sociality, demarcating the blurry boundaries between play and aggression in a society characterized by despotism.

Other reasons for the absence of a congruency effect in chimpanzees may be related to limitations to our cross-modal priming design. As facial and bodily cues were shown separately, subjects may have processed the two stimuli as isolated events. However, this explanation is unlikely, since chimpanzees have been shown to match auditory with visual emotion expressions in another cross-modal paradigm presenting stimuli on separate screens with a 1–2 s delay (24). From an evolutionary standpoint (i.e. applying the principle of evolutionary parsimony), chimpanzees should, as humans, be able to infer affective information from bodily cues. Indeed, a study using naturalistic social stimuli revealed that chimpanzees attend more strongly toward threatening bodily scenes compared to other contexts (i.e. even when lacking facial and vocal cues), suggesting that bodily movements do carry affective information (19). The explanation that the chimpanzees' strong *fear-related* attention bias could have masked the priming effects thus appears to be most plausible. Although chimpanzees may be able to infer emotional states from contextual (25) and bodily cues (19), the effect may be attenuated and overshadowed in spontaneous eye-tracking priming paradigms by an overarching attention bias toward threatening emotional content.

Indeed, one may argue that static images of facial expressions—as used in our study—are unnatural and thus hinder the chimpanzees from connecting the facial expressions to the corresponding bodily primers. Although we cannot test this with this data, there are several reasons to refute this explanation as a drawback of our study. First, there is ample evidence that chimpanzees can recognize and remember emotions from facial and bodily expressions in the form of static images across various paradigms, including computerized touch screen experiments (22) as well as match-to-sample tests (24, 25). Crucially, chimpanzees can recognize and discriminate facial expressions from static images *and* match these to video material (24, 25). With no prior training, chimpanzees can spontaneously match emotional video scenes with static images of facial expressions based on emotional meaning, even when the images are in gray scale (25). In another experiment, in which chimpanzees viewed dynamic scenes of whole-body expressions (with facial and vocal information masked), chimpanzees viewed scenes of agonistic scenes longer than scenes of neutrality, excitement, and playfulness, regardless of the pixel-level properties, playback speeds, number of individuals in clips, or presentation order of the videos (19). These lines of evidence suggest that the physical properties such as clip quality and dynamic nature do not appear to hinder chimpanzees' perception of emotions from static images. In addition, our chimpanzees underwent a scramble test before preceding to the actual experiment, in which we assessed their attention levels toward static images of food items (either presented in destructed form, i.e. scrambled in pixels vs. as normal image). The test (see Text S7 and Table S6.1) clearly demonstrates that chimpanzees look longer toward food items that were not scrambled rather than scrambled, indicating they perceive images based on content rather than basic-level physical properties. In sum, we believe there is sufficient evidence to reason that the chimpanzees in our study could comprehend the content from the images and the videos, providing the necessary capacity to draw associations across image and video stimuli. To expand the evidence of the current study, we nevertheless advocate for future studies to manipulate the design of such an experiment and instead use (i) dynamic short-clips of facial expressions, and (ii) as mentioned above, presenting whole-body video scenes alongside cross-modal stimuli (e.g. emotional vocalizations congruent or incongruent with the whole-body scenes).

Although our sample size of chimpanzees reflects the standards of other studies using eye-tracking in apes (14), it is also important to note that our conclusions are drawn from a relatively small sample, which is not uncommon for comparative research of this kind with primates: there are substantial challenges of conducting computerized eye-tracking experiments with great apes, which remains still a new area of research (14). To date, eye-tracking technology has been designed for human participants, which makes it harder to obtain high-quality calibration results for nonhuman primates, who have different ocular morphologies and attention spans for stimuli presented on screens (14). Due to these methodological constrains, in computerized tasks as such researchers are usually only able to test a limited sample of apes (14). We initially had eight chimpanzees engage with the eye-tracker, but as data quality was a priority, we only retained the data from the six individuals we could fully calibrate, to ensure to a comparable degree to human calibration standards. This high standard is important for generating valid comparisons with human data, though does place constraints on possible sample sizes. To deal with this issue, we have used Bayesian regression models, which are known to be much more robust than usual frequentist models in dealing with small samples (82). Kruschke et al. (82) state that in analyses with small samples, frequentist approaches can easily lead to type I/II errors as the small sample violates assumptions for obtaining an accurate *P-*value. In contrast, Bayesian posterior distributions, such as provided in our results, reveal estimations about uncertainty in parameter estimates. In Bayesian statistics “sample size does not affect inference method” (p. 740, Ref. (82)). To solidify the evidence and draw generalized claims, we still advocate future studies to repeat our study in other chimpanzee communities.

Though more research is needed, our findings overall point to a less biased form of emotion processing in humans as compared to chimpanzees. We believe that the reported discrepancies may be related to differences in the species' social structures. Chimpanzees have been documented to be more despotic than humans, whilst humans are assumed to have lived most of human history as hunter gatherers in self-organized, mainly egalitarian societies before Neolithic cultures emerged (Refs. (83, 84), but see Ref. (85)). In chimpanzee societies, which frequently involve nonlethal physical aggression (86), and authoritative, despotic leadership—though this can vary among groups (87), it can be detrimental to fail in accurately deciphering emotional states, notably if these are related to aggression. Though speculative, for humans, life in more egalitarian structures may have enabled more flexible affective communication strategies, facilitating the emergence of context-related emotion processing with greater influence of bodily (or general contextual) cues to disambiguate affective information.

Finally, in terms of interspecies emotion perception, the relevant priming effect in humans disappeared when humans viewed chimpanzee stimuli. Only when viewing the *resting* primers of chimpanzees, yet not the *play* primers, human participants looked faster at images showing play faces compared to bared-teeth expressions. Humans might have associated a soothing, or affiliative, emotional state with chimpanzee *resting* primers (i.e. which may likewise explain an attenuation of the threat-related bias in chimpanzees following *resting* primers). However, humans may have had difficulties in disentangling *play* and *fear* primers, perhaps because both types of scenes are fast-paced, dynamic and contain movements of slapping or hitting. At the same time, as our sample of human participants did not represent a sample of nonhuman primate communication experts, our results represent a rare and spontaneous interspecies assessment of humans' perception of chimpanzee emotional expressions. A lack of understanding and expertise in judging great ape emotion signals could have prevented the priming effects. Indeed, research has shown that primate experts are better at judging primate social behavior compared to nonexperts (50). Notably though, human adults performed better at such interpretation tasks compared to children, pointing to an effect of learning experience in cross-species emotion recognition (88). Yet another study showed that humans (untrained in judging ape behavior) pay more attention to static images showing emotional expressions of chimpanzees compared to neutral versions (20). In line with the attention patterns, humans also rated emotional expressions of chimpanzees in static images as non-neutral, but they struggled to accurately judge the *valence* of specific emotion categories such as anger or fear (20). Putting our results into context, it thus appears that humans can broadly distinguish between calm versus emotionally charged emotion cues of apes (see also Ref. (20)). However, humans seem to have difficulties in spontaneously associating ape emotion cues from different modalities, or to determine their social function and valence, when lacking experience in judging ape behavior (88). Given effects of experience and learning, it would be interesting to repeat our study with chimpanzee experts or with young children who may be entirely naïve (e.g. see Ref. (88)).

## Conclusion

A key finding is that humans, more so than chimpanzees, associate specific facial emotion expressions with bodily emotion cues, even when no other information is provided. The emotional bodily scenes in humans, yet not in chimpanzees, had a priming effect, insofar as they steered attention to the corresponding facial expression (*fear* primer –*fearful* facial expression; *play* primer—*smiling* facial expression). Chimpanzees, in contrast, were highly fixated on *bared-teeth* over *neutral* facial expressions, regardless of the bodily primer. These findings suggest a more biased style of emotion perception in chimpanzees compared to humans, with greater focus towards threat or *fear*-related facial expressions and potentially reduced levels of cross-modal integration. Finally, although humans struggled in associating facial expressions with corresponding bodily cues of their close chimpanzee relatives, they were nonetheless able to broadly discern between neutral (affiliative and calm) and affective (dynamic and emotionally charged) scenes, which we find corroborates the current literature.

## Supplementary Material

pgae012_Supplementary_Data

## Data Availability

All data and relevant R code are freely accessible on figshare.com under https://doi.org/10.6084/m9.figshare.22561213.v2.
