# Supplementary material for: Perceptual integration of bodily and facial emotion cues in chimpanzees and humans
Source: PNAS Nexus. 2024 Jan 18;3(2):pgae012. doi: 10.1093/pnasnexus/pgae012 (PMC10855020; doi:10.1093/pnasnexus/pgae012)
Supplement: pgae012_Supplementary_Data [file pgae012_supplementary_data.zip › Supplementary_Materials_Heesen et al.docx]

***Supplementary material***

**Perceptual integration of bodily and facial emotion cues in chimpanzees and humans**

Raphaela Heesen^1^*^&^, Yena Kim^2&^, Mariska E. Kret^2,3^, Zanna Clay^1^*

^1^ Department of Psychology, Durham University, UK

^2^ Institute of Psychology, Cognitive Psychology Unit, Leiden University, Netherlands

^3^The Leiden Institute for Brain and Cognition, Leiden University, Netherlands

*Joint corresponding authors: [heesenr1@gmail.com](mailto:heesenr1@gmail.com); [zanna.e.clay@durham.ac.uk](mailto:zanna.e.clay@durham.ac.uk)

^&^ Joint first authorship

**Text S1.** Study site and group

Basel zoo comprises a building with six interconnected inside lodges (233 m^2^), which are further connected with two outside ones (477 m^2^). The chimpanzees could freely roam between the lodges, which include climbing structures, ropes, puzzle boxes and other enrichment items. The group, which contained 14 members (details Table S1) at the time, was fed six times a day, with salad, vegetables, fruits, eggs, and specialized primate pellets.

**Text S2.** Basel zoo eye-tracking setup

The eye-tracker was built into a small cage which was connected to the chimpanzee large enclosure through a 1cm thick transparent plexiglass panel, through with the chimpanzees could watch the stimuli on the screen while their eye-gaze was being assessed. The small cage was kept locked during the experiment for safety in case the plexiglass would break. The centre of the plexiglass panel contained a drinking nozzle through which the chimpanzees could suck juice throughout their session to minimize the chimpanzees’ head movements during the experiments. The juice was diluted and contained sugar free sirup; it was administered through a small tube connected to a catheter outside the enclosure; the juice flow was steady to keep the amount consistent throughout the experiment, controlled by the experimenter who was located outside the small cage and the chimpanzee enclosure in the nearby corridor. An extended laptop was connected to the HP webcam as well as the eye-tracker, such that the experimenter was able to manually administer trials and monitor the participant’s behaviour and gaze. The testing took place in one of their indoor compartments such that the apes were still visible to the visitors. While watching the screen, the participants were seated on a small platform, where they could either hold on to a rope or small handle next to the apparatus. The chimpanzees were never separated from group members during the experiment, thus tested in a social group setting.

**Text S3.** Further details on design, stimuli content, selection, and validation

Experiments 1-3 entailed four types of primers. Three showed digitized video clips of unfamiliar conspecifics’ bodily behaviour (facial and vocal information masked) in either *playful* (humans: playful scenes where people laugh, chimpanzees: social play), *fearful* (humans: scenes where people get scared by others in the form of scary pranks, chimpanzees: conflict between individuals), or neutral *resting* scenes (humans and chimpanzees standing, slowly walking, or calmly resting in proximity of others). One additional neutral *fish tank* control included a fish tank scene (used for both species). We primed participants with these scenes and then tested their initial orientation and sustained attention towards the corresponding static images of either *fearful* or *playful* facial expressions. These facial expressions of emotion (“emotion images”) were presented adjacent to neutral facial expressions of the *same* individual. Congruent trials involved images of facial expressions matching the previous scene (e.g., humans: a *fearful* face following a *fear* primer; chimpanzees: a *bared-teeth* facial expression following a *fear* primer) and incongruent trials involved images of facial expressions mismatching the previous scene (e.g., humans: a *fearful* face following the *play* primer; chimpanzees: a *bared-teeth* facial expression following a *play* primer). In chimpanzees, previous research has demonstrated that the play face [muscle action units “AUs” 12+25+26: 1] reliably occurs in social play [2–6], whilst the bared-teeth display [AUs 10+12+16+25: 1] is frequently connected with agonistic situations and conflict, signalling submission towards dominants [5]. In humans, “enjoyment” smiles, also called Duchenne smiles (characterized by the activation of the *orbicularis oculi* muscle AU06 in combination with the *zygomatic major* muscle pulling the lips AU12 [7;8]) are produced across a wide range of contexts, but are predominantly produced in response to positive, genuinely amusing stimuli [see for review 9]. Fear faces (involving combinations of three to six different AUs, such as the inner eye-brow raiser AU1 and outer eye-brow raiser AU2, opening of eyes through the upper lid raiser AU5, and open mouth through lip stretcher AU20 and lip parting AU25 [8]) are primarily produced in scary or potentially threatening situations where the signaller repels a potential attack via their own submission or imminent retreat [10]. These facial expressions, as described above, reliably accompany the bodily movements depicted across the different valence contexts, which is why they were chosen for this experiment.

The chimpanzee stimuli set (video scenes and images of facial expressions) presented in this study were selected based on criteria of highest intensity/clearest valence by the great ape expert researchers who authored this study. Most of the stimuli presented were already validated in other emotion studies prior to this study by other primate emotion experts (e.g., Lisa Parr).

For the human stimuli set, the 90 edited videos were rated by five experts on human emotion communication using a Qualtrics survey, based on category (7 choices: anger, fear, happiness, surprise, sadness, disgust, and neutral), valence (7-point Likert scale: 1 being negative, 4 being neutral, 7 being positive), and intensity (7-point Likert scale: 1 being not at all intense, 4 being moderately intense, 7 being extremely intense) of the emotion depicted in the scene, see for results Table S3.

**Text S4.** Details on video stimuli content and preparation

*Experiment 1: Chimpanzees viewing chimpanzee stimuli.* We focused on dyadic scenes without additional bystanders. If bystanders occurred in the original videos, they were blurred in Adobe Premier Pro CC 2020 (version 14.3). Faces of chimpanzee subjects in the videos were masked using the inbuilt face tracking function and a gaussian blur (oval mask) in Adobe Premier Pro. Vocalizations were also masked by removing sound from the scenes. The scenes were picked with a preference for no vegetation to ensure good visibility of the chimpanzees’ bodies. Scenes were always cut in ways to ensure movements were not cut midway; for instance, if a chimpanzee slapped the ground in a conflict, the movement was carried out until the end, thus the video cut after the movement had ended. Nonetheless, the 3sec video duration ways always respected. Scenes were cut also in ways to depict actors in the centre of the scene, to ensure attention to be relatively controlled across scenes. Scenes depicted captive chimpanzees (except one) filmed predominantly in an outdoor enclosure to avoid fences and blurry windows and ensure good light conditions; for videos shot in the inside enclosures, the videos had to be high quality and no window glare or mesh obstructed the view. Conspecific scenes contained activity-specific bodily cues such as movement speed, acceleration, body tension or jerkiness. Example videos can be found in movies s1-s4.

*Experiment 2: Humans viewing human stimuli.* As for chimpanzees, scenes were cut in ways to depict actors in the centre of the scene, to ensure attention to be relatively controlled across scenes. Additional adjustments of the resolution (1280 x 1024 pixels) and masking faces of the actors were made using a built-in tracking function with Gaussian Blur. If emotion eliciting objects or bystanders were visible in the original videos, they were blurred as well. All the sounds in the priming scenes were removed. *Fear* primers were all from scary prank videos on YouTube, showing mostly two individuals (mean = 2.4, SD = 0.82), ranging from one to four, reacting to a fear-eliciting stimulus. The reactions typically included freezing and/or fleeing. *Play* primers were either from funny prank videos or spontaneous laughing videos on YouTube or free stock videos on the internet (e.g., Pexels). In most cases, except for one (three individuals in a scene), two individuals were laughing out loud with rhythmic body movements. All the *neutral social* priming scenes were from YouTube, showing mostly two individuals (*mean* = 2.2, *SD* = 0.52), ranging from two to four, walking the street or sitting on a bench. Example videos can be found in movies s6.

**Text S5.** Details on image pair content and preparation

*Experiment 1: Chimpanzees viewing chimpanzee stimuli.* Adjacently matched images were controlled for a similar head positioning and gaze direction. Images showed the entire head of chimpanzees, including a small part of the neck or shoulder as the opening of the mouth often stretched widely. To adjust individuals’ gaze, head direction, and upper body positioning on the image pairs we used the stamp function in GIMP (version 2.10.22) to match features like hair sticking off at certain points of the body, or to remove spots on the actors’ bodies caused through sunlight. Each face image was scaled to 400 × 330 pixels in GIMP and matched in resolution using 240 pixels per inch. Once images were prepared, we cropped the shape of the individuals face and upper body from the former background, added an alpha channel and removed the background. We accumulated all these images in a folder to compute luminance values. All images were tightly controlled for average luminance values using MatLab (MathWorks) by first computing each images’ average luminance values and manually adjusting the images to match them as closely as possible. This resulted in a controlled luminance level of *mean* = 0.32, *range* = 0.31-0.33, *SD* = 0.01. We subjectively matched images for contrast, black level, and exposure. Single images were presented against a background of a greyish cement wall typical for captive holding areas and then paired side by side on against a black background (1280 x 1024 pixels), see examples in movies s1-s4 and Fig. 1 in main manuscript.

Similarly, for *scrambled* and *nonscrambled* images, we scaled images to 400 × 330 pixels; food items were presented against a plain white background. *Scrambled* and *nonscrambled* images were presented side-by-side on against a black background (1280 x 1024 pixels), see example movie s5. We scrambled the pixels of each image showing food items using MatLab.

*Experiment 2: Humans viewing human stimuli.* We selected Caucasian faces to minimize potential effects of ethnicity. For the Chicago face database, we selected 106 faces from 52 actors (26 females) based on the highest suitability, intensity, and valence for each category of the faces. For the Radboud face database, we selected 72 faces from 36 actors faces (18 females) based on the highest category agreement, authenticity, intensity, and valence for each category of the faces. Each face image was cropped from the tip of the head until the bottom of the jaw in 400 x 330 pixels and matched in resolution using 240 pixels per inch in Adobe Photoshop (version 22.2) to standardize size the face across images. For the same reason, we replaced the background colour of the face to grey using a build-in Remove Background function in Adobe Photoshop. No luminance adjustment was necessary as they were already luminance-controlled faces. For both the image pair types, the emotional face was placed next to the neutral face of the same individual with a 375-pixel gap in a black background (1280 x 1024 pixels).

**Text S6.** Calibration procedure and results for chimpanzees

For calibration, the participants looked at small videos of changing fruits or primate faces (if needed with an alerting sound) at random locations on the screen, always either left or right side and bottom or top of the screen, until the researcher could see the eye-gaze properly fixated on the corresponding spot on their laptop (Notebook Captiva NH50_70RA). We repeated calibration as much as it was needed to achieve maximum accuracy; this meant that the gaze was calibrated until each participant reached an average calibration accuracy (both eyes) < 0.4°, precision *SD* < 0.4° and 0% data loss (see Table for Text S6 below). These calibration results are acceptable and, in some cases, even reach the level of human testing requirements, which requires an average calibration accuracy (both eyes) < 0.8°, precision *SD* < 0.5° and ideally 0% data loss as suggested by Tobii Technology AB in 2012 [11].

**Table for Text S6.** Calibration results for chimpanzee participants. Results are total averages including the left and right eye results.

| **Participant** | **Accuracy** | **Precision (RMS)** | **Precision (SD)** | **Data loss** |
| --- | --- | --- | --- | --- |
| Benga | 0.27° | 0.20° | 0.16° | 0% |
| Kume | 0.11° | 0.13° | 0.10° | 0% |
| Lazima | 0.36° | 0.16° | 0.16° | 0% |
| Obaye | 0.05° | 0.15° | 0.09° | 0% |
| Obuasi | 0.29° | 0.37° | 0.36° | 0% |
| Ponima | 0.08° | 0.17° | 0.08° | 0% |

******Note***.** RMS = Root mean square; SD = Standard deviation.

**Text S7.** Attention validation test results

Chimpanzees looked at the original images substantially longer than towards *scrambled* images (*median* = 0.81, *MAD =* 0.03, 89% *CrI* [0.76, 0.86], *pd* = 100 %), see also Table S6.1 attention validation model.

**Text S8.** Descriptive results for general attention

Out of 3 sec presentation time, chimpanzees on average looked at conspecific images for 1.34 sec (*SD* = 0.67), and humans on average looked at conspecific images for 2.58 sec (*SD* = 0.36) and chimpanzee images for 2.43 sec (*SD* = 0.58).

**Table S1.** Demographic information of chimpanzee members in the group at Basel Zoo in 2021. Chimpanzees who participated in the testing are marked in italic bold.

| **ID** | **birthdate** | **age in years (2021)** | **sex** |
| --- | --- | --- | --- |
| ***Benga*** | ***1979-09-17*** | ***42*** | ***F*** |
| Colebe | 2005-07-12 | 16 | M |
| Fifi | 1993-05-12 | 28 | F |
| Garissa | 2009-04-06 | 12 | F |
| Jacky* | 1968-01-01 | 54 | F |
| Kitoko | 1993-05-20 | 28 | F |
| ***Kume*** | ***2003-10-04*** | ***18*** | ***M*** |
| ***Lazima*** | ***2014-07-29*** | ***7*** | ***F*** |
| ***Obaye*** | ***2017-09-27*** | ***4*** | ***M*** |
| ***Obuasi*** | ***2017-12-26*** | ***4*** | ***F*** |
| ***Ponima*** | ***2018-05-22*** | ***3*** | ***F*** |
| Qisenge | 2019-08-13 | 2 | F |
| Xindra | 1975-10-23 | 46 | F |

********Note.* Jacky’s age was roughly estimated, as she was not born in a zoo and rescued from private owners.

**Table S2.** Sex and age characteristics of chimpanzee actors in the primers of Experiment 1. A = Adult; S = Subadult; M = Male; F = Female.

| **Negative** |  |  |  |  |
| --- | --- | --- | --- | --- |
|  | **M-M** | **F-F** | **M-F** | *Total* |
| **A-A** | 6 | - | 1 | *7* |
| **A-S** | 1 | 2 | 4 | *7* |
| **S-S** | - | 5 | 1 | *6* |
| *Total* | *7* | *7* | *6* | *20* |
|  |  |  |  |  |
| **Positive** |  |  |  |  |
|  | **M-M** | **F-F** | **M-F** | *Total* |
| **A-A** | 6 | 1 | - | *7* |
| **A-S** | - | - | 7 | *7* |
| **S-S** | 1 | 5 | - | *6* |
| *Total* | *7* | *6* | *7* | *20* |
|  |  |  |  |  |
| **Neutral** |  |  |  |  |
|  | **M-M** | **F-F** | **M-F** | *Total* |
| **A-A** | 7 | - | - | *7* |
| **A-S** | - | - | 7 | *7* |
| **S-S** | - | 6 | - | *6* |
| *Total* | *7* | *6* | *7* | *20* |

**Table S3.**Average (*SD*) of expert ratings (*N*=5) on valence, intensity, authenticity, and category for final primer stimuli of Experiment 2.

| **Primer** | **Valence**  *(1 = negative, 4 = neutral, 7 = positive)* | **Intensity**  *(1 = not at all intense, 7 = extremely intense)* | **Authenticity**  *(1 = not at all realistic, 4 = moderately realistic, 7 = extremely realistic)* | **Category***  *(0-5 raters agreed)* |
| --- | --- | --- | --- | --- |
| Fear | 1.94(0.46) | 5.38(0.88) | 6.39(0.28) | 3.95(0.69) |
| Play | 6.12(0.48) | 4.83(0.82) | 6.24(0.54) | 4.95(0.22) |
| Resting | 3.97(0.07) | 2.08(0.34) | 5.3(0.37) | 5(0) |

*Category refers to the number of raters who selected the same, relevant category for primer videos.

**Table S4.** Gender and age characteristics of adult human actors in the primers of Experiment 2. A = Adult; T = Teenager; M = Man; W = Woman.

| **Negative** |  |  |  |
| --- | --- | --- | --- |
| **M-M** | **W-W** | **M-W** | *Total* |
| 4 | 11 | 5 | *20* |
|  |  |  |  |
| **Positive** |  |  |  |
| **M-M** | **F-F** | **M-F** | *Total* |
| 7 | 4 | 9 | *20* |
|  |  |  |  |
| **Neutral** |  |  |  |
| **M-M** | **F-F** | **M-F** | *Total* |
| 7 | 4 | 9 | *20* |

**Table S5.** Bayesian point estimates of emotion biases towards emotion images across primer conditions. *Fish tank* primers, which indicate basic emotion biases, are noted in bold *(*note that basic emotion bias is fulfilled if median >0.5 and 89% CrI* ***not*** *centering at 0.5).*

S5.1: Experiment 1.

| ***TFF_EmoBinary_*** (Initial orientation) |  |  |  |  |
| --- | --- | --- | --- | --- |
| *Primer condition* | *Emotion image* | *Median* | *L-89%* | *U-89%* |
| **Fish tank** | **Bared-teeth** | **0.68** | **0.60** | **0.77** |
| Fear | Bared-teeth | 0.66 | 0.56 | 0.75 |
| Resting | Bared-teeth | 0.60 | 0.50 | 0.70 |
| Play | Bared-teeth | 0.68 | 0.58 | 0.77 |
| **Fish tank** | **Play face** | **0.52** | **0.42** | **0.62** |
| Fear | Play face | 0.58 | 0.48 | 0.69 |
| Resting | Play face | 0.57 | 0.46 | 0.67 |
| Play | Play face | 0.56 | 0.46 | 0.66 |

| ***TFD_EmoProp_*** (Sustained attention) |  |  |  |  |
| --- | --- | --- | --- | --- |
| *Primer condition* | *Emotion image* | *Median* | *L-89%* | *U-89%* |
| **Fish tank** | **Bared-teeth** | **0.62** | **0.57** | **0.66** |
| Fear | Bared-teeth | 0.59 | 0.54 | 0.63 |
| Resting | Bared-teeth | 0.57 | 0.53 | 0.62 |
| Play | Bared-teeth | 0.60 | 0.55 | 0.64 |
| **Fish tank** | **Play face** | **0.50** | **0.45** | **0.55** |
| Fear | Play face | 0.52 | 0.46 | 0.57 |
| Resting | Play face | 0.55 | 0.51 | 0.61 |
| Play | Play face | 0.51 | 0.46 | 0.56 |

S5.2: Experiment 2.

| ***TFF_EmoBinary_*** (Initial orientation) |  |  |  |  |
| --- | --- | --- | --- | --- |
| *Primer condition* | *Emotion image* | *Median* | *L-89%* | *U-89%* |
| **Fish tank** | **Fearful** | **0.54** | **0.47** | **0.60** |
| Fear | Fearful | 0.53 | 0.45 | 0.60 |
| Play | Fearful | 0.54 | 0.47 | 0.61 |
| Resting | Fearful | 0.56 | 0.49 | 0.63 |
| **Fish tank** | **Smiling** | **0.54** | **0.47** | **0.61** |
| Fear | Smiling | 0.53 | 0.46 | 0.60 |
| Play | Smiling | 0.58 | 0.51 | 0.64 |
| Resting | Smiling | 0.55 | 0.49 | 0.62 |

| ***TFD_EmoProp_*** (Sustained attention) |  |  |  |  |
| --- | --- | --- | --- | --- |
| *Primer condition* | *Emotion image* | *Median* | *L-89%* | *U-89%* |
| **Fish tank** | **Fearful** | **0.54** | **0.52** | **0.56** |
| Fear | Fearful | 0.55 | 0.54 | 0.57 |
| Play | Fearful | 0.54 | 0.52 | 0.55 |
| Resting | Fearful | 0.53 | 0.51 | 0.55 |
| **Fish tank** | **Smiling** | **0.54** | **0.52** | **0.56** |
| Fear | Smiling | 0.53 | 0.51 | 0.55 |
| Play | Smiling | 0.55 | 0.54 | 0.57 |
| Resting | Smiling | 0.55 | 0.53 | 0.56 |

S5.3: Experiment 3.

| ***TFF_EmoBinary_*** (Initial orientation) |  |  |  |  |
| --- | --- | --- | --- | --- |
| *Primer condition* | *Emotion image* | *Median* | *L-89%* | *U-89%* |
| **Fish tank** | **Bared-teeth** | **0.53** | **0.48** | **0.58** |
| Fear | Bared-teeth | 0.58 | 0.53 | 0.63 |
| Play | Bared-teeth | 0.52 | 0.47 | 0.57 |
| Neutral | Bared-teeth | 0.56 | 0.51 | 0.61 |
| **Fish tank** | **Play face** | **0.54** | **0.49** | **0.59** |
| Fear | Play face | 0.50 | 0.45 | 0.55 |
| Play | Play face | 0.52 | 0.47 | 0.57 |
| Resting | Play face | 0.59 | 0.54 | 0.64 |

| ***TFD_EmoProp_*** (Sustained attention) |  |  |  |  |
| --- | --- | --- | --- | --- |
| *Primer condition* | *Emotion image* | *Median* | *L-89%* | *U-89%* |
| **Fish tank** | **Bared-teeth** | **0.52** | **0.50** | **0.55** |
| Fear | Bared-teeth | 0.55 | 0.52 | 0.57 |
| Play | Bared-teeth | 0.52 | 0.50 | 0.55 |
| Resting | Bared-teeth | 0.49 | 0.47 | 0.52 |
| **Fish tank** | **Play face** | **0.51** | **0.48** | **0.53** |
| Fear | Play face | 0.53 | 0.50 | 0.55 |
| Play | Play face | 0.51 | 0.49 | 0.54 |
| Resting | Play face | 0.48 | 0.46 | 0.51 |

***Abbreviation:** *L/U-89%* = Lower and upper 89% Credible Intervals

**Table S6.** Bayesian model summaries. Substantial effects of predictor variables in bold. Random effects in italics. *

S6.1: Attention validation model for chimpanzees.

| ***TFD_EmoProp_* (N = 142)** |  |  |  |  |  |  |  |
| --- | --- | --- | --- | --- | --- | --- | --- |
|  | *Bulk_ESS* | *Tail_ESS* | *R*ˆ | *b* | *S.D.* | *L-89%* | *U-89%* |
| Intercept | 6858 | 6713 | 1.00 | 1.45 | 0.21 | 1.10 | 1.76 |
| phi Intercept (*scamble* vs. *nonscramble*) | 5741 | 6463 | 1.00 | 2.74 | 0.61 | 1.79 | 3.65 |
| zoi Intercept | 8136 | 7949 | 1.00 | -1.15 | 0.54 | -2.03 | -0.32 |
| coi Intercept | 8324 | 9169 | 1.00 | 3.40 | 1.71 | 0.66 | 6.17 |
| **Participant ID** |  |  |  |  |  |  |  |
| sd(Intercept) | 4435 | 4445 | 1.00 | 0.37 | 0.22 | 0.12 | 0.75 |
| sd(phi Intercept) | 5021 | 7632 | 1.00 | 1.25 | 0.58 | 0.59 | 2.30 |
| sd(zoi Intercept) | 4043 | 6010 | 1.00 | 0.63 | 0.51 | 0.06 | 1.54 |
| sd(coi Intercept) | 5705 | 7549 | 1.00 | 3.26 | 3.62 | 0.35 | 9.21 |
| **Participant ID: Session Number** |  |  |  |  |  |  |  |
| sd(Intercept) | 3947 | 6101 | 1.00 | 0.12 | 0.09 | 0.01 | 0.27 |
| sd(phi Intercept) | 4484 | 6287 | 1.00 | 0.31 | 0.23 | 0.03 | 0.74 |
| sd(zoi Intercept) | 2497 | 2617 | 1.00 | 1.05 | 0.53 | 0.22 | 1.94 |
| sd(coi Intercept) | 9147 | 5961 | 1.00 | 0.99 | 0.85 | 0.07 | 2.59 |
| **Image ID** |  |  |  |  |  |  |  |
| sd(Intercept) | 3306 | 2914 | 1.00 | 0.41 | 0.10 | 0.25 | 0.58 |
| sd(phi Intercept) | 2928 | 5315 | 1.00 | 0.41 | 0.30 | 0.04 | 0.95 |
| sd(zoi Intercept) | 4483 | 6114 | 1.00 | 1.48 | 0.51 | 0.77 | 2.37 |
| sd(coi Intercept) | 6788 | 6335 | 1.00 | 1.63 | 1.27 | 0.16 | 3.92 |

S6.2: Experiment 1. Reference level in parentheses.

| **Model 1. *TFF_EmoBinary_* (N = 797)** |  |  |  |  |  |  |  |  |
| --- | --- | --- | --- | --- | --- | --- | --- | --- |
|  | *Bulk_ESS* | *Tail_ESS* | *R*ˆ | *b* | *S.D.* | *L-89%* | *U-89%* | *pd* |
| Intercept | 9213 | 9939 | 1.00 | 0.81 | 0.25 | 0.41 | 1.21 | 99.97% |
| *Fear* primer [*Fish tank* primer] | 10688 | 9551 | 1.00 | -0.11 | 0.31 | -0.61 | 0.38 | 63.82% |
| *Resting* primer [*Fish tank* primer] | 9680 | 9601 | 1.00 | -0.36 | 0.32 | -0.87 | 0.15 | 86.82% |
| *Play* primer [*Fish tank* primer] | 12351 | 9348 | 1.00 | -0.02 | 0.30 | -0.50 | 0.46 | 52.59% |
| **Image play face [bared-teeth]** | **8587** | **8828** | **1.00** | **-0.69** | **0.31** | **-1.18** | **-0.20** | **98.80**% |
| Trial repetition yes [no] | 26413 | 9206 | 1.00 | -0.08 | 0.20 | -0.40 | 0.25 | 64.62% |
| *Fear* primer [*Fish tank* primer] x Image play face [bared-teeth] | 11354 | 9398 | 1.00 | 0.36 | 0.40 | -0.28 | 1.00 | 81.15% |
| *Resting* primer [*Fish tank* primer] x Image play face [bared-teeth] | 9875 | 9112 | 1.00 | 0.55 | 0.42 | -0.11 | 1.21 | 90.24% |
| *Play* primer [*Fish tank* primer] x Image play face [bared-teeth] | 11023 | 9974 | 1.00 | 0.17 | 0.39 | -0.46 | 0.79 | 66.56% |
| *Image ID* | *3641* | *5282* | *1.00* | *0.66* | *0.14* | *0.44* | *0.88* | *-* |
| *Subject ID* | *6517* | *7317* | *1.00* | *0.12* | *0.11* | *0.01* | *0.31* | *-* |
| *Subject ID: Session Number* | *3059* | *5524* | *1.00* | *0.21* | *0.15* | *0.02* | *0.48* | *-* |
| *Primer ID* | *3500* | *5398* | *1.00* | *0.18* | *0.13* | *0.02* | *0.42* | *-* |
|  |  |  |  |  |  |  |  |  |
| **Model 2. *TFD_EmoProp_* (N = 797)** |  |  |  |  |  |  |  |  |
|  | *Bulk_ESS* | *Tail_ESS* | *R*ˆ | *b* | *S.D.* | *L-89%* | *U-89%* | *pd* |
| Intercept | 5599 | 8177 | 1.00 | 0.51 | 0.12 | 0.32 | 0.71 | 100% |
| *Fear* primer [*Fish tank* primer] | 5939 | 7641 | 1.00 | -0.13 | 0.14 | -0.35 | 0.09 | 82.59% |
| *Resting* primer [*Fish tank* primer] | 5595 | 7768 | 1.00 | -0.18 | 0.15 | -0.42 | 0.06 | 88.55% |
| *Play* primer [*Fish tank* primer] | 6265 | 8133 | 1.00 | -0.09 | 0.14 | -0.31 | 0.13 | 74.36% |
| **Image play face [bared-teeth]** | **4953** | **7298** | **1.00** | **-0.47** | **0.14** | **-0.69** | **-0.25** | **99.99**% |
| Trial repetition yes [no] | 15762 | 10033 | 1.00 | -0.06 | 0.08 | -0.19 | 0.07 | 78.15% |
| *Fear* primer [*Fish tank* primer] x Image play face [bared-teeth] | 6175 | 8342 | 1.00 | 0.18 | 0.17 | -0.10 | 0.45 | 84.48% |
| ***Resting* primer [*Fish tank* primer] x Image play face [bared-teeth**] | **5656** | **8158** | **1.00** | **0.39** | **0.19** | **0.08** | **0.69** | **97.95**% |
| *Play* primer [*Fish tank* primer] x Image play face [bared-teeth] | 6582 | 8665 | 1.00 | 0.11 | 0.17 | -0.16 | 0.38 | 74.23% |
| *Image pair ID* | *4454* | *6496* | *1.00* | *0.33* | *0.05* | *0.26* | *0.41* | *-* |
| *Subject ID* | *3558* | *4775* | *1.00* | *0.09* | *0.07* | *0.01* | *0.20* | *-* |
| *Subject ID: Session Number* | *2229* | *2121* | *1.00* | *0.21* | *0.06* | *0.11* | *0.29* | *-* |
| *Primer ID* | *2193* | *2170* | *1.00* | *0.22* | *0.06* | *0.13* | *0.31* | *-* |

S6.3: Experiment 2. Reference level in parentheses.

| **Model 3. *TFF_EmoBinary_* (N = 3791)** | *Bulk_ESS* | *Tail_ESS* | *R*ˆ | *b* | *S.D.* | *L-89%* | *U-89%* | *pd* |
| --- | --- | --- | --- | --- | --- | --- | --- | --- |
| Intercept | 4998 | 7132 | 1.00 | 0.12 | 0.17 | -0.16 | 0.39 | 74.72*%* |
| *Fear* primer [*Fish tank* primer] | 4872 | 7238 | 1.00 | -0.05 | 0.24 | -0.44 | 0.34 | 57.97*%* |
| *Resting* primer [*Fish tank* primer] | 4971 | 7317 | 1.00 | 0.08 | 0.24 | -0.30 | 0.46 | 61.85*%* |
| *Play* primer [*Fish tank* primer] | 5165 | 8155 | 1.00 | -0.01 | 0.24 | -0.39 | 0.37 | 51.86*%* |
| Image smiling face [fearful] | 4728 | 6635 | 1.00 | 0.00 | 0.23 | -0.36 | 0.36 | 49.81*%* |
| Gender man [woman] | 20047 | 9793 | 1.00 | 0.07 | 0.09 | -0.07 | 0.21 | 78.97*%* |
| *Fear* primer [*Fish tank* primer] x Image smiling face [fearful] | 5091 | 7444 | 1.00 | 0.02 | 0.32 | -0.49 | 0.53 | 53.15*%* |
| *Resting* primer [*Fish tank* primer] x Image smiling face [fearful] | 4850 | 7244 | 1.00 | -0.01 | 0.31 | -0.51 | 0.49 | 51.69*%* |
| *Play* primer [*Fish tank* primer] x Image smiling face [fearful] | 5058 | 7590 | 1.00 | 0.18 | 0.32 | -0.33 | 0.69 | 70.66*%* |
| *Image ID* | *4695* | *7857* | *1.00* | *0.66* | *0.06* | *0.57* | *0.76* | *-* |
| *Participant ID* | *5064* | *6362* | *1.00* | *0.07* | *0.05* | *0.01* | *0.15* | *-* |
| *Participant ID: Session Number* | *1144* | *3465* | *1.00* | *0.19* | *0.11* | *0.03* | *0.37* | *-* |
| *Primer ID* | *3434* | *4816* | *1.00* | *0.07* | *0.06* | *0.01* | *0.18* | *-* |
|  |  |  |  |  |  |  |  |  |
| **Model 4. *TFD_EmoProp_* (N = 3791)** | *Bulk_ESS* | *Tail_ESS* | *R*ˆ | *b* | *S.D.* | *L-89%* | *U-89%* | *pd* |
| Intercept | 3393 | 5296 | 1.00 | 0.17 | 0.05 | 0.09 | 0.25 | 99.96% |
| *Fear* primer [*Fish tank* primer] | 5055 | 7428 | 1.00 | 0.07 | 0.05 | -0.01 | 0.15 | 92.25% |
| *Resting* primer [*Fish tank* primer] | 5073 | 7396 | 1.00 | -0.04 | 0.05 | -0.12 | 0.04 | 54.76% |
| *Play* primer [*Fish tank* primer] | 5006 | 8024 | 1.00 | -0.01 | 0.05 | -0.09 | 0.07 | 78.51% |
| Image smiling face [fearful] | 3972 | 6579 | 1.00 | 0.02 | 0.05 | -0.06 | 0.09 | 65.73% |
| Gender man [woman] | 3001 | 5500 | 1.00 | -0.05 | 0.07 | -0.16 | 0.06 | 77.09% |
| ***Fear* primer [*Fish tank* primer] x Image smiling face [fearful]** | **4748** | **7238** | **1.00** | **-0.12** | **0.07** | **-0.23** | **-0.01** | **95.63**% |
| *Resting* primer [*Fish tank* primer] x Image smiling face [fearful] | 4909 | 7733 | 1.00 | 0.06 | 0.07 | -0.05 | 0.17 | 79.31% |
| *Play* primer [*Fish tank* primer] x Image smiling face [fearful] | 4922 | 7660 | 1.00 | 0.06 | 0.07 | -0.05 | 0.17 | 80.52% |
| *Image pair ID* | *1887* | *3110* | *1.00* | *0.04* | *0.02* | *0.01* | *0.08* | *-* |
| *Participant ID* | *3727* | *6222* | *1.00* | *0.14* | *0.03* | *0.10* | *0.18* | *-* |
| *Participant ID: Session Number* | *848* | *742* | *1.00* | *0.11* | *0.04* | *0.04* | *0.16* | *-* |
| *Primer ID* | *3026* | *5001* | *1.00* | *0.03* | *0.02* | *0.00* | *0.06* | *-* |

S6.4: Experiment 3. Reference level in parentheses.

| **Model 5. *TFF_EmoBinary_* (N = 3559)** | *Bulk_ESS* | *Tail_ESS* | *R*ˆ | *b* | *S.D.* | *L-89%* | *U-89%* | *pd* |  |
| --- | --- | --- | --- | --- | --- | --- | --- | --- | --- |
| Intercept | 6552 | 8195 | 1.00 | 0.13 | 0.12 | -0.06 | 0.33 | 85.73% |  |
| *Fear* primer [*Fish tank* primer] | 6932 | 9180 | 1.00 | 0.19 | 0.17 | -0.07 | 0.46 | 87.60% |  |
| *Resting* primer [*Fish tank* primer] | 7141 | 8421 | 1.00 | 0.11 | 0.17 | -0.15 | 0.37 | 74.90% |  |
| *Play* primer [*Fish tank* primer] | 7251 | 8838 | 1.00 | -0.05 | 0.16 | -0.31 | 0.21 | 62.14% |  |
| Image play face [bared-teeth] | 6587 | 8374 | 1.00 | 0.04 | 0.14 | -0.18 | 0.26 | 59.92% |  |
| Gender man [woman] | 11797 | 9119 | 1.00 | -0.02 | 0.11 | -0.19 | 0.15 | 56.64% |  |
| ***Fear* primer [*Fish tank* primer]** **x Image play face [bared-teeth]** | **7299** | **8346** | **1.00** | **-0.35** | **0.19** | **-0.66** | **-0.04** | **96.43**% |  |
| *Resting* primer [*Fish tank* primer] x Image play face [bared-teeth] | 7550 | 9124 | 1.00 | 0.10 | 0.20 | -0.22 | 0.42 | 68.52% |  |
| *Play* primer [*Fish tank* primer] x Image play face [bared-teeth] | 8127 | 9275 | 1.00 | -0.03 | 0.19 | -0.34 | 0.27 | 55.73% |  |
| *Image ID* | *2688* | *2155* | *1.00* | *0.11* | *0.07* | *0.01* | *0.22* | *-* |  |
| *Participant ID* | *3383* | *4005* | *1.00* | *0.12* | *0.06* | *0.03* | *0.21* | *-* |  |
| *Participant ID: Session Number* | *6991* | *5801* | *1.00* | *0.04* | *0.03* | *0.00* | *0.11* | *-* |  |
| *Primer ID* | *5286* | *6925* | *1.00* | *0.30* | *0.05* | *0.22* | *0.39* | *-* |  |
|  |  |  |  |  |  |  |  |  |  |
| **Model 6. *TFD_EmoProp_* (N = 3715)** | *Bulk_ESS* | *Tail_ESS* | *R*ˆ | *b* | *S.D.* | *L-89%* | *U-89%* | *pd* |  |
| Intercept | 2876 | 5461 | 1.00 | 0.14 | 0.06 | 0.04 | 0.24 | 98.58% |  |
| *Fear* primer [*Fish tank* primer] | 5892 | 8147 | 1.00 | 0.09 | 0.06 | -0.01 | 0.17 | 93.14% |  |
| ***Resting* primer [*Fish tank* primer]** | **5136** | **8103** | **1.00** | **-0.13** | **0.06** | **-0.22** | **-0.03** | **98.39%** |  |
| *Play* primer [*Fish tank* primer] | 6376 | 8588 | 1.00 | 0.00 | 0.06 | -0.09 | 0.09 | 52.66% |  |
| Image play face [bared-teeth] | 4123 | 6652 | 1.00 | -0.07 | 0.06 | -0.16 | 0.03 | 86.77% |  |
| Gender man [woman] | 3672 | 5388 | 1.00 | -0.10 | 0.11 | -0.27 | 0.06 | 83.02% |  |
| *Fear* primer [*Fish tank* primer] x Image play face [bared-teeth] | 5479 | 7373 | 1.00 | -0.00 | 0.08 | -0.13 | 0.12 | 51.02% |  |
| *Resting* primer [*Fish tank* primer] x Image play face [bared-teeth] | 5043 | 7347 | 1.00 | 0.02 | 0.08 | -0.10 | 0.16 | 61.52% |  |
| *Play* primer [*Fish tank* primer] x Image play face [bared-teeth] | 5426 | 7704 | 1.00 | 0.03 | 0.08 | -0.10 | 0.15 | 63.14% |  |
| *Image pair ID* | *3571* | *4735* | *1.00* | *0.12* | *0.02* | *0.09* | *0.15* | *-* |  |
| *Participant ID* | *3652* | *6512* | *1.00* | *0.20* | *0.04* | *0.15* | *0.26* | *-* |  |
| *Participant ID: Session Number* | *937* | *1821* | *1.00* | *0.09* | *0.04* | *0.01* | *0.15* | - |  |
| *Primer ID* | *2089* | *4516* | *1.00* | *0.04* | *0.03* | *0.01* | *0.09* | - |  |

* **Abbreviations**: *phi* = precision of the beta distribution; *zoi* = zero-one inflation probability, *coi* = conditional one inflation probability; *b*= Estimated mean of the posterior distribution; *S.D.*= Standard deviation of the posterior distribution; *L/U-89%* = Lower and upper 89% Credible Intervals; *R*ˆ=R hat value, provides information about the convergence of the MCMC algorithm - if larger than 1.1, chains have not converged and model is not accurate; *Bulk/Tail_ESS*= number of effective sample sizes in either bulk or tail of posterior distribution; *pd* = probability of direction, i.e., an index of effect existence, see for details <https://easystats.github.io/bayestestR/articles/probability_of_direction.html>

**Table S7.** Contrasts of existing effects using estimated marginal means (EMMs) for Models 1-6. *

S7.1: Experiment 1. Substantial effects marked in bold.

| **Model 1. *TFF_EmoBinary_*** |  |  |  |  |
| --- | --- | --- | --- | --- |
| *Primer:Image – Primer:Image (reference level)* | *Median* | *MAD* | *89% CrI* | *pd* |
| Fish tank:Bared-teeth – Fear:Bared-teeth | 0.12 | 0.31 | [-0.40, 0.59] | 64.28% |
| Fish tank:Bared-teeth - Resting:Bared-teeth | 0.36 | 0.32 | [-0.14, 0.87] | 87.19% |
| Fish tank:Bared-teeth - Play:Bared-teeth | 0.02 | 0.3 | [-0.46, 0.50] | 52.62% |
| **Fish tank**:**Bared-teeth - Fish tank**:**Play face** | **0.69** | **0.31** | **[ 0.21, 1.19]** | **98.90%** |
| Fish tank:Bared-teeth - Fear:Play face | 0.45 | 0.36 | [-0.13, 0.99] | 89.28% |
| Fish tank:Bared-teeth - Resting:Play face | 0.51 | 0.36 | [-0.07, 1.07] | 92.03% |
| Fish tank:Bared-teeth – Play:Play face | 0.54 | 0.34 | [-0.01, 1.10] | 93.83% |
| Fear:Bared-teeth - Resting:Bared-teeth | 0.25 | 0.32 | [-0.28, 0.74] | 77.79% |
| Fear:Bared-teeth – Play:Bared-teeth | -0.09 | 0.35 | [-0.67, 0.46] | 60.48% |
| **Fear**:**Bared-teeth - Fish tank**:**Play face** | **0.58** | **0.36** | **[ 0.00, 1.14]** | **94.70%** |
| Fear:Bared-teeth - Fear: Play face | 0.33 | 0.35 | [-0.22, 0.89] | 82.66% |
| Fear:Bared-teeth - Resting: Play face | 0.39 | 0.36 | [-0.19, 0.97] | 86.48% |
| Fear:Bared-teeth – Play:Play face | 0.43 | 0.37 | [-0.18, 0.99] | 87.57% |
| Resting:Bared-teeth – Play:Bared-teeth | -0.34 | 0.33 | [-0.88, 0.17] | 85.37% |
| Resting:Bared-teeth - Fish tank:Play face | 0.33 | 0.35 | [-0.22, 0.92] | 82.35% |
| Resting:Bared-teeth - Fear: Play face | 0.08 | 0.37 | [-0.50, 0.66] | 59.08% |
| Resting:Bared-teeth - Resting: Play face | 0.15 | 0.35 | [-0.41, 0.69] | 66.57% |
| Resting:Bared-teeth – Play:Play face | 0.17 | 0.35 | [-0.39, 0.75] | 68.86% |
| **Play**:**Bared-teeth - Fish tank**:**Play face** | **0.67** | **0.36** | **[ 0.10, 1.23]** | **97.30%** |
| Play:Bared-teeth - Fear: Play face | 0.42 | 0.36 | [-0.17, 0.98] | 87.66% |
| Play:Bared-teeth - Resting:Play face | 0.49 | 0.37 | [-0.08, 1.07] | 90.92% |
| Play:Bared-teeth – Play:Play face | 0.52 | 0.34 | [-0.01, 1.07] | 93.58% |
| Fish tank:Play face - Fear: Play face | -0.25 | 0.32 | [-0.76, 0.27] | 78.36% |
| Fish tank:Play face – Resting:Play face | -0.18 | 0.34 | [-0.72, 0.34] | 70.48% |
| Fish tank:Play face – Play:Play face | -0.15 | 0.31 | [-0.65, 0.34] | 69.07% |
| Fear:Play face - Resting: Play face | 0.06 | 0.33 | [-0.50, 0.54] | 57.56% |
| Fear:Play face – Play:Play face | 0.09 | 0.35 | [-0.47, 0.65] | 60.77% |
| Resting:Play face – Play:Play face | 0.03 | 0.32 | [-0.48, 0.56] | 54.11% |
|  |  |  |  |  |
| **Model 2. *TFD_EmoProp_*** |  |  |  |  |
| *Primer:Image – Primer:Image (reference level)* | *Median* | *MAD* | *89% CrI* | *pd* |
| Fish tank:Bared-teeth – Fear:Bared-teeth | 0.13 | 0.14 | [-0.09, 0.35] | 82.88% |
| Fish tank:Bared-teeth - Resting:Bared-teeth | 0.18 | 0.15 | [-0.04, 0.43] | 88.85% |
| Fish tank:Bared-teeth - Play:Bared-teeth | 0.09 | 0.14 | [-0.13, 0.31] | 74.67% |
| **Fish tank**:**Bared-teeth - Fish tank**:**Play face** | **0.46** | **0.14** | **[ 0.25, 0.69]** | **99.95%** |
| **Fish tank:Bared-teeth - Fear:Play face** | **0.42** | **0.16** | **[ 0.17, 0.68]** | **99.56%** |
| **Fish tank:Bared-teeth - Resting:Play face** | **0.26** | **0.16** | **[ 0.00, 0.52]** | **94.75%** |
| **Fish tank:Bared-teeth – Play:Play face** | **0.45** | **0.16** | **[ 0.19, 0.71]** | **99.63%** |
| Fear:Bared-teeth - Resting:Bared-teeth | 0.05 | 0.14 | [-0.18, 0.27] | 64.29% |
| Fear:Bared-teeth – Play:Bared-teeth | -0.04 | 0.15 | [-0.29, 0.19] | 60.61% |
| **Fear**:**Bared-teeth - Fish tank**:**Play face** | **0.34** | **0.16** | **[ 0.08, 0.59]** | **98.17%** |
| **Fear:Bared-teeth - Fear:Play face** | **0.29** | **0.15** | **[ 0.05, 0.52]** | **97.62%** |
| Fear:Bared-teeth - Resting:Play face | 0.13 | 0.16 | [-0.12, 0.40] | 78.83% |
| **Fear:Bared-teeth – Play:Play face** | **0.31** | **0.16** | **[ 0.05, 0.57]** | 97.32% |
| Resting:Bared-teeth – Play:Bared-teeth | -0.09 | 0.14 | [-0.30, 0.14] | 74.14% |
| **Resting:Bared-teeth - Fish tank:Play face** | **0.29** | **0.16** | **[ 0.03, 0.53]** | **96.23%** |
| Resting:Bared-teeth - Fear:Play face | 0.24 | 0.16 | [-0.01, 0.51] | 92.72% |
| Resting:Bared-teeth - Resting:Play face | 0.08 | 0.15 | [-0.16, 0.31] | 70.76% |
| **Resting:Bared-teeth – Play:Play face** | **0.26** | **0.16** | **[ 0.00, 0.53]** | **94.66%** |
| **Play:Bared-teeth - Fish tank:Play face** | **0.37** | **0.15** | **[ 0.12, 0.63]** | **99.12%** |
| **Play:Bared-teeth - Fear:Play face** | **0.33** | **0.16** | **[ 0.08, 0.58]** | **97.97%** |
| Play:Bared-teeth - Resting:Play face | 0.17 | 0.16 | [-0.09, 0.42] | 86.02% |
| **Play:Bared-teeth – Play:Play face** | **0.35** | **0.14** | **[ 0.12, 0.58]** | **99.14%** |
| Fish tank:Play face - Fear:Play face | -0.05 | 0.14 | [-0.26, 0.19] | 62.74% |
| Fish tank:Play face – Resting:Play face | -0.2 | 0.15 | [-0.44, 0.04] | 91.10% |
| Fish tank:Play face – Play:Play face | -0.02 | 0.14 | [-0.26, 0.20] | 55.72% |
| Fear:Play face - Resting:Play face | -0.16 | 0.14 | [-0.40, 0.06] | 86.69% |
| Fear:Play face – Play:Play face | 0.02 | 0.16 | [-0.21, 0.29] | 56.32% |
| Resting:Play face – Play:Play face | 0.18 | 0.15 | [-0.04, 0.43] | 89.67% |

S7.2: Experiment 2. Substantial effects marked in bold.

| **Model 3. *TFF_EmoBinary_*** | | |  | |
| --- | --- | --- | --- | --- |
| *Primer:Image – Primer:Image (reference level)* | *Median* | *MAD* | *89% CrI* | *pd* |
| Fish tank:Fearful - Fear:Fearful | 0.05 | 0.24 | [-0.33, 0.44] | 58.43% |
| Fish tank:Fearful - Play:Fearful | 0.01 | 0.24 | [-0.37, 0.39] | 52.10% |
| Fish tank:Fearful - Resting:Fearful | -0.07 | 0.24 | [-0.45, 0.31] | 62.22% |
| Fish tank:Fearful - Fish tank:Smiling | 0.00 | 0.22 | [-0.36, 0.36] | 50.13% |
| Fish tank:Fearful - Fear:Smiling | 0.03 | 0.24 | [-0.37, 0.40] | 54.11% |
| Fish tank:Fearful - Play:Smiling | -0.17 | 0.25 | [-0.57, 0.22] | 74.98% |
| Fish tank:Fearful - Resting:Smiling | -0.07 | 0.24 | [-0.47, 0.31] | 60.80% |
| Fear:Fearful - Play:Fearful | -0.04 | 0.25 | [-0.45, 0.35] | 56.57% |
| Fear:Fearful - Resting:Fearful | -0.13 | 0.25 | [-0.54, 0.27] | 69.36% |
| Fear:Fearful - Fish tank:Smiling | -0.05 | 0.25 | [-0.47, 0.34] | 57.73% |
| Fear:Fearful - Fear:Smiling | -0.03 | 0.24 | [-0.42, 0.36] | 54.07% |
| Fear:Fearful - Play:Smiling | -0.22 | 0.25 | [-0.64, 0.18] | 80.98% |
| Fear:Fearful - Resting:Smiling | -0.12 | 0.24 | [-0.52, 0.27] | 68.57% |
| Play:Fearful - Resting:Fearful | -0.09 | 0.25 | [-0.49, 0.31] | 63.43% |
| Play:Fearful - Fish tank:Smiling | -0.02 | 0.25 | [-0.41, 0.38] | 52.37% |
| Play:Fearful - Fear:Smiling | 0.01 | 0.25 | [-0.38, 0.40] | 52.01% |
| Play:Fearful - Play:Smiling | -0.18 | 0.24 | [-0.58, 0.20] | 76.84% |
| Play:Fearful - Resting:Smiling | -0.08 | 0.24 | [-0.47, 0.31] | 62.78% |
| Resting:Fearful - Fish tank:Smiling | 0.08 | 0.25 | [-0.32, 0.47] | 62.18% |
| Resting:Fearful - Fear:Smiling | 0.1 | 0.24 | [-0.30, 0.49] | 66.03% |
| Resting:Fearful - Play:Smiling | -0.09 | 0.25 | [-0.51, 0.28] | 63.75% |
| Resting:Fearful - Resting:Smiling | 0.01 | 0.23 | [-0.36, 0.39] | 52.08% |
| Fish tank:Smiling - Fear:Smiling | 0.03 | 0.24 | [-0.34, 0.43] | 54.79% |
| Fish tank:Smiling - Play:Smiling | -0.17 | 0.24 | [-0.54, 0.23] | 74.97% |
| Fish tank:Smiling - Resting:Smiling | -0.06 | 0.24 | [-0.45, 0.30] | 60.37% |
| Fear:Smiling - Play:Smiling | -0.19 | 0.25 | [-0.57, 0.21] | 78.50% |
| Fear:Smiling - Resting:Smiling | -0.09 | 0.24 | [-0.48, 0.28] | 65.28% |
| Play:Smiling - Resting:Smiling | 0.1 | 0.24 | [-0.28, 0.48] | 66.13% |
| **Model 4. *TFD_EmoProp_*** |  |  |  |  |
| *Primer:Image – Primer:Image (reference level)* | *Median* | *MAD* | *89% CrI* | *pd* |
| Fish tank:Fearful - Fear:Fearful | -0.07 | 0.05 | [-0.15, 0.01] | 92.54% |
| Fish tank:Fearful - Play:Fearful | 0.01 | 0.05 | [-0.08, 0.08] | 54.88% |
| Fish tank:Fearful - Resting:Fearful | 0.04 | 0.05 | [-0.04, 0.12] | 78.97% |
| Fish tank:Fearful - Fish tank:Smiling | -0.02 | 0.05 | [-0.09, 0.06] | 65.92% |
| Fish tank:Fearful - Fear:Smiling | 0.03 | 0.05 | [-0.05, 0.10] | 71.06% |
| Fish tank:Fearful - Play:Smiling | -0.07 | 0.05 | [-0.14, 0.01] | 92.03% |
| Fish tank:Fearful - Resting:Smiling | -0.04 | 0.05 | [-0.12, 0.04] | 78.37% |
| **Fear:Fearful - Play:Fearful** | **0.08** | **0.05** | **[ 0.00, 0.16]** | **94.40%** |
| **Fear:Fearful - Resting:Fearful** | **0.11** | **0.05** | **[ 0.03, 0.19]** | **98.67%** |
| Fear:Fearful - Fish tank:Smiling | 0.05 | 0.05 | [-0.02, 0.13] | 86.64% |
| **Fear:Fearful - Fear:Smiling** | **0.1** | **0.05** | **[ 0.02, 0.17]** | **98.22%** |
| Fear:Fearful - Play:Smiling | 0.00 | 0.05 | [-0.08, 0.08] | 53.32% |
| Fear:Fearful - Resting:Smiling | 0.03 | 0.05 | [-0.05, 0.11] | 76.15% |
| Play:Fearful - Resting:Fearful | 0.03 | 0.05 | [-0.05, 0.11] | 75.12% |
| Play:Fearful - Fish tank:Smiling | -0.03 | 0.05 | [-0.10, 0.05] | 70.56% |
| Play:Fearful - Fear:Smiling | 0.02 | 0.05 | [-0.05, 0.10] | 66.56% |
| Play:Fearful - Play:Smiling | -0.08 | 0.05 | [-0.15, 0.00] | 94.05% |
| Play:Fearful - Resting:Smiling | -0.05 | 0.05 | [-0.12, 0.03] | 82.12% |
| Resting:Fearful - Fish tank:Smiling | -0.06 | 0.05 | [-0.13, 0.02] | 88.78% |
| Resting:Fearful - Fear:Smiling | -0.01 | 0.05 | [-0.09, 0.07] | 60.17% |
| **Resting:Fearful - Play:Smiling** | **-0.11** | **0.05** | **[-0.19, -0.03]** | **98.77%** |
| Resting:Fearful - Resting:Smiling | -0.08 | 0.05 | [-0.15, 0.00] | 94.90% |
| Fish tank:Smiling - Fear:Smiling | 0.05 | 0.05 | [-0.03, 0.12] | 84.08% |
| Fish tank:Smiling - Play:Smiling | -0.05 | 0.05 | [-0.12, 0.02] | 85.59% |
| Fish tank:Smiling - Resting:Smiling | -0.02 | 0.05 | [-0.09, 0.05] | 65.91% |
| **Fear:Smiling - Play:Smiling** | **-0.1** | **0.05** | **[-0.17, -0.02]** | **97.65%** |
| Fear:Smiling - Resting:Smiling | -0.07 | 0.05 | [-0.14, 0.01] | 91.78% |
| Play:Smiling - Resting:Smiling | 0.03 | 0.05 | [-0.04, 0.11] | 74.43% |

S7.3: Experiment 3. Substantial effects marked in bold.

| **Model 5. *TFF_EmoBinary_*** | | | |  |
| --- | --- | --- | --- | --- |
| *Primer:Image – Primer:Image (reference level)* | *Median* | *MAD* | *89% CrI* | *pd* |
| Fish tank:Bared-teeth – Fear:Bared-teeth | -0.19 | 0.17 | [-0.45, 0.07] | 87.98% |
| Fish tank:Bared-teeth – Play:Bared-teeth | 0.05 | 0.16 | [-0.21, 0.31] | 62.31% |
| Fish tank:Bared-teeth – Resting:Bared-teeth | -0.11 | 0.16 | [-0.37, 0.16] | 75.40% |
| Fish tank:Bared-teeth - Fish tank:Play face | -0.04 | 0.14 | [-0.26, 0.18] | 59.93% |
| Fish tank:Bared-teeth – Fear:Play face | 0.12 | 0.17 | [-0.15, 0.39] | 76.15% |
| Fish tank:Bared-teeth – Play:Play face | 0.04 | 0.16 | [-0.22, 0.31] | 60.15% |
| Fish tank: Bared-teeth – Resting:Play face | -0.25 | 0.17 | [-0.52, 0.04] | 92.44% |
| Fear:Bared-teeth – Play:Bared-teeth | 0.24 | 0.17 | [-0.02, 0.52] | 92.54% |
| Fear:Bared-teeth – Resting:Bared-teeth | 0.08 | 0.17 | [-0.18, 0.36] | 68.75% |
| Fear:Bared-teeth – Fish tank:Play face | 0.16 | 0.17 | [-0.11, 0.43] | 81.88% |
| **Fear:Bared-teeth – Fear:Play face** | **0.31** | **0.14** | **[ 0.10, 0.55]** | **98.70%** |
| Fear:Bared-teeth – Play:Play face | 0.24 | 0.17 | [-0.04, 0.51] | 92.01% |
| Fear:Bared-teeth – Resting:Play face | -0.06 | 0.18 | [-0.34, 0.22] | 61.99% |
| Play:Bared-teeth – Resting:Bared-teeth | -0.16 | 0.17 | [-0.43, 0.10] | 83.81% |
| Play:Bared-teeth – Fish tank:Play face | -0.09 | 0.17 | [-0.36, 0.18] | 69.37% |
| Play:Bared-teeth – Fear:Play face | 0.07 | 0.17 | [-0.20, 0.34] | 65.83% |
| Play:Bared-teeth – Play:Play face | -0.01 | 0.14 | [-0.22, 0.22] | 52.37% |
| **Play:Bared-teeth – Resting:Play face** | **-0.3** | **0.18** | **[-0.57, -0.01]** | **95.72%** |
| Resting:Bared-teeth – Fish tank:Play face | 0.08 | 0.17 | [-0.19, 0.35] | 67.49% |
| Resting:Bared-teeth – Fear:Play face | 0.23 | 0.17 | [-0.05, 0.50] | 91.12% |
| Resting:Bared-teeth – Play:Play face | 0.16 | 0.17 | [-0.12, 0.43] | 82.07% |
| Resting:Bared-teeth – Resting:Play face | -0.13 | 0.15 | [-0.37, 0.10] | 82.35% |
| Fish tank:Play face- Fear:Play face | 0.16 | 0.17 | [-0.11, 0.43] | 82.00% |
| Fish tank:Play face- Play:Play face | 0.08 | 0.17 | [-0.18, 0.36] | 68.26% |
| Fish tank:Play face – Resting:Play face | -0.21 | 0.17 | [-0.49, 0.06] | 88.52% |
| Fear:Play face – Play:Play face | -0.08 | 0.17 | [-0.34, 0.20] | 67.42% |
| **Fear:Play face – Resting:Play face** | **-0.37** | **0.18** | **[-0.64, -0.08]** | **98.26%** |
| **Play:Play face – Resting:Play face** | **-0.29** | **0.17** | **[-0.57, -0.02]** | **95.52%** |
|  |  |  |  |  |
| **Model 6. *TFD_EmoProp_*** |  |  |  |  |
| *Primer: Image – Primer:Image (reference level)* | *Median* | *MAD* | *89% CrI* |  |
| Fish tank:Bared-teeth – Fear:Bared-teeth | -0.09 | 0.06 | [-0.17, 0.01] | 93.38% |
| Fish tank:Bared-teeth – Play:Bared-teeth | -0.00 | 0.06 | [-0.09, 0.09] | 52.98% |
| **Fish tank:Bared-teeth – Resting:Bared-teeth** | **0.12** | **0.06** | **[ 0.03, 0.22]** | **98.49%** |
| Fish tank:Bared-teeth - Fish tank:Play face | 0.07 | 0.06 | [-0.03, 0.16] | 87.07% |
| Fish tank:Bared-teeth – Fear:Play face | -0.02 | 0.06 | [-0.12, 0.08] | 60.88% |
| Fish tank:Bared-teeth – Play:Play face | 0.04 | 0.06 | [-0.06, 0.14] | 72.70% |
| **Fish tank: Bared-teeth – Resting:Play face** | **0.17** | **0.06** | **[ 0.07, 0.27]** | **99.62%** |
| Fear:Bared-teeth – Play:Bared-teeth | 0.08 | 0.06 | [-0.01, 0.18] | 91.63% |
| **Fear:Bared-teeth – Resting:Bared-teeth** | **0.21** | **0.06** | **[ 0.12, 0.30]** | **100%** |
| **Fear:Bared-teeth – Fish tank:Play face** | **0.15** | **0.06** | **[ 0.06, 0.25]** | **99.38%** |
| Fear:Bared-teeth – Fear:Play face | 0.07 | 0.06 | [-0.03, 0.16] | 87.68% |
| **Fear:Bared-teeth – Play:Play face** | **0.12** | **0.06** | **[ 0.03, 0.22]** | **97.82%** |
| **Fear:Bared-teeth – Resting:Play face** | **0.25** | **0.06** | **[ 0.16, 0.35]** | **99.99%** |
| **Play:Bared-teeth – Resting:Bared-teeth** | **0.13** | **0.05** | **[ 0.04, 0.22]** | **99.23%** |
| Play:Bared-teeth – Fish tank:Play face | 0.07 | 0.06 | [-0.03, 0.17] | 88.30% |
| Play:Bared-teeth – Fear:Play face | -0.01 | 0.06 | [-0.11, 0.09] | 58.45% |
| Play:Bared-teeth – Play:Play face | 0.04 | 0.06 | [-0.05, 0.13] | 76.28% |
| **Play:Bared-teeth – Resting:Play face** | **0.17** | **0.06** | **[ 0.07, 0.27]** | **99.78%** |
| Resting:Bared-teeth – Fish tank:Play face | -0.06 | 0.06 | [-0.16, 0.03] | 83.45% |
| **Resting:Bared-teeth – Fear:Play face** | **-0.14** | **0.06** | **[-0.24, -0.05]** | **98.98%** |
| Resting:Bared-teeth – Play:Play face | -0.09 | 0.06 | [-0.18, 0.01] | 93.12% |
| Resting:Bared-teeth – Resting:Play face | 0.04 | 0.06 | [-0.05, 0.14] | 76.06% |
| Fish tank:Play face- Fear:Play face | -0.08 | 0.06 | [-0.18, 0.01] | 92.91% |
| Fish tank:Play face- Play:Play face | -0.03 | 0.06 | [-0.12, 0.06] | 69.93% |
| **Fish tank:Play face – Resting:Play face** | **0.1** | **0.06** | **[ 0.00, 0.20]** | **94.82%** |
| Fear:Play face – Play:Play face | 0.05 | 0.06 | [-0.05, 0.15] | 81.34% |
| **Fear:Play face – Resting:Play face** | **0.18** | **0.06** | **[ 0.10, 0.28]** | **99.97%** |
| **Play:Play face – Resting:Play face** | **0.13** | **0.06** | **[ 0.04, 0.23]** | **98.78%** |

* **Abbreviations:** *MAD* = Median absolute deviation; 89% *CrI* = 89% Credible Interval to report the limits of the highest density intervals (*HDIs*) of the estimates.

**Table S8.** Leave-one-out cross-validation (LOOIC) comparisons.

1. ***Adding “bared-teeth intensity” as factor.***

| **Model** | **LOOIC** | **ELPD difference** | **SE difference** |
| --- | --- | --- | --- |
| Model *TFF_EmoBinary_* (reduced) | 517.7 | -0.4 | 0.5 |
| Model *TFF_EmoBinary_* (full) | 518.4 |  |  |
|  |  |  |  |
| Model *TFD_EmoProp_* (reduced) | 65.5 | -0.4 | 0.5 |
| Model *TFD_EmoProp_* (full) | 66.2 |  |  |
|  |  |  |  |

1. ***Adding “age” as factor. Models 1 and 2 represent those from Table S6.***

| **Model** | **LOOIC** | **ELPD difference** | **SE difference** |
| --- | --- | --- | --- |
| Model *1* (reduced) | 1054.8 | -1.0 | 0.7 |
| Model *1* (full) | 1056.8 |  |  |
|  |  |  |  |
| Model 2 (reduced) | 1054.8 | -544.9 | 29.7 |
| Model 2 (full) | -35.1 |  |  |
|  |  |  |  |

1. ***Adding “sex” as factor. Models 1 and 2 represent those from Table S6.***

| **Model** | **LOOIC** | **ELPD difference** | **SE difference** |
| --- | --- | --- | --- |
| Model *1* (reduced) | 1054.8 | -0.4 | 1.0 |
| Model *1* (full) | 1055.6 |  |  |
|  |  |  |  |
| Model 2 (reduced) | 1054.8 | -546.7 | 29.5 |
| Model 2 (full) | -38.6 |  |  |
|  |  |  |  |

#### Note. LOOIC comparing model fit including or excluding the respective variables above. The first listed model version (“reduced”: excluding the respective variable) is compared against secondary model versions (“full”: including the respective variable). The difference in the expected log predictive densities (ELPD) were negative, suggesting that model accuracies do not improve by adding these respective variables, for which reason only the reduced models are presented in the main paper. For problematic Pareto k values, we set IC comparisons to RELOO=TRUE. Note that the models likewise did not reveal robust effects of intensity/age/sex on TFF_EmoBinary_ and TFD_EmoProp,_ which can be found in our R code based on model summaries, saved as .rds files.

**Supplementary movies**

**Movie S1.** Example video of a female chimpanzee watching a *play* primer with a subsequent image pair comprising a *neutral* and *play face* expression.

**Movie S2.** Example video of a female chimpanzee watching a *fear* primer with a subsequent image pair comprising a *neutral* and *bared-teeth* expression.

**Movie S3.** Example video of a male chimpanzee watching a *resting* primer with a subsequent image pair comprising a *neutral* and *bared-teeth* expression.

**Movie S4.** Example video of a female chimpanzee watching a *fish tank* primer with a subsequent image pair comprising a *neutral* and *play face* expression.

**Movie S5.** Example video of a male chimpanzee watching a *scrambled* vs. *nonscrambled* image.

**Movie S6**. Example video of a human participant watching all four primers in a session.

**References of the supplementary materials**

1. Parr L.A., Waller B.M., Vick S.J., Bard K.A.. 2007 Classifying chimpanzee facial expressions using muscle action. *Emotion* **7**, 172–81. (doi:10.1037/1528-3542.7.1.172)

2. Demuru E, Ferrari PF, Palagi E. 2015 Emotionality and intentionality in bonobo playful communication. *Anim Cogn* **18**, 333–344. (doi:10.1007/s10071-014-0804-6)

3. Palagi E. 2008 Sharing the motivation to play: the use of signals in adult bonobos. *Anim Behav* **75**, 887–896. (doi:10.1016/J.ANBEHAV.2007.07.016)

4. Palagi E. 2006 Social play in bonobos (*Pan paniscus*) and chimpanzees (*Pan troglodytes*): Implicationsfor natural social systems and interindividual relationships. *Am J Phys Anthropol* **129**, 418–426. (doi:10.1002/ajpa.20289)

5. Parr L, Cohen M, Waal F de. 2005 Influence of social context on the use of blended and graded facial displays in chimpanzees. *Int J Primatol* **26**, 73–103. (doi:10.1007/s10764-005-0724-z)

6. van Hooff JA. 1972 A comparative approach to the phylogeny of laughter and smiling. In R.A. Hinde (ed.), *Non-verbal communication.* Cambridge: Cambridge University Press.

7. Gunnery SD, Hall JA, Ruben MA. 2013 The Deliberate Duchenne Smile: Individual Differences in Expressive Control. *J Nonverbal Behav* **37**, 29–41. (doi:10.1007/S10919-012-0139-4/TABLES/4)

8. Ekman P, Friesen W, Hager JC. 2002 *Facial Action Coding System: The manual*. Salt Lake City, USA: Research Nexus.

9. Kret ME, Prochazkova E, Sterck EHM, Clay Z. 2020 Emotional expressions in human and non-human great apes. *Neurosci Biobehav Rev* **115**, 378–395. (doi:10.1016/j.neubiorev.2020.01.027)

10. Crivelli C, Fridlund AJ. 2018 Facial Displays Are Tools for Social Influence. *Trends Cogn Sci* **22**, 388–399. (doi:10.1016/J.TICS.2018.02.006)

11. Tobii Technology AB. 2012 Test specification: Accuracy and precision test method for remote eye trackers.
